# The Use of Human Mesenchymal Stem Cells as Therapeutic Agents for the *in vivo* Treatment of Immune-Related Diseases: A Systematic Review

**DOI:** 10.3389/fimmu.2018.02056

**Published:** 2018-09-11

**Authors:** Alessander Leyendecker Jr., Carla Cristina Gomes Pinheiro, Mariane Tami Amano, Daniela Franco Bueno

**Affiliations:** Hospital Sírio Libanês, São Paulo, Brazil

**Keywords:** mesenchymal stem cell, immunomodulation, immunomodulatory therapy, immune-related diseases, autoimmune diseases, inflammation, inflammatory diseases, neurologic diseases

## Abstract

**Background:** One of the greatest challenges for medicine is to find a safe and effective treatment for immune-related diseases. However, due to the low efficacy of the treatment available and the occurrence of serious adverse effects, many groups are currently searching for alternatives to the traditional therapy. In this regard, the use of human mesenchymal stem cells (hMSCs) represents a great promise for the treatment of a variety of immune-related diseases due to their potent immunomodulatory properties. The main objective of this study is, therefore, to present and summarize, through a systematic review of the literature, *in vivo* studies in which the efficacy of the administration of hMSCs for the treatment of immune-related diseases was evaluated.

**Methods:** The article search was conducted in PubMed/MEDLINE, Scopus and Web of Science databases. Original research articles assessing the therapeutic potential of hMSCs administration for the *in vivo* treatment immune-related diseases, published from 1984 to December 2017, were selected and evaluated.

**Results:** A total of 132 manuscripts formed the basis of this systematic review. Most of the studies analyzed reported positive results after hMSCs administration. Clinical effects commonly observed include an increase in the survival rates and a reduction in the severity and incidence of the immune-related diseases studied. In addition, hMSCs administration resulted in an inhibition in the proliferation and activation of CD19^+^ B cells, CD4^+^ Th1 and Th17 cells, CD8^+^ T cells, NK cells, macrophages, monocytes, and neutrophils. The clonal expansion of both Bregs and Tregs cells, however, was stimulated. Administration of hMSCs also resulted in a reduction in the levels of pro-inflammatory cytokines such as IFN-γ, TNF-α, IL-1, IL-2, IL-12, and IL-17 and in an increase in the levels of immunoregulatory cytokines such as IL-4, IL-10, and IL-13.

**Conclusions:** The results obtained in this study open new avenues for the treatment of immune-related diseases through the administration of hMSCs and emphasize the importance of the conduction of further studies in this area.

## Introduction

### Rationale

Autoimmune diseases result from defects in the mechanisms of immunological tolerance, culminating in the activation of cellular and humoral mechanisms of the immune response against self-antigens (1, 2). As a result, in autoimmune diseases, a failure occurs in the body's ability to differentiate cells from the body from foreign cells. These diseases may be restricted to a particular organ or be systemic (3). Examples of autoimmune diseases include type I diabetes mellitus, myasthenia gravis, multiple sclerosis, systemic lupus erythematosus and systemic sclerosis. The mechanism and causes of the occurrence of autoimmune diseases are still not well-understood, however, it is believed that the origin of the majority of these diseases is multifactorial, in which both genetic and environmental factors are involved (4–6). Due to the possible occurrence of bone marrow toxicity, caused by the immunosuppressive regimen currently applied in the conventional treatment (7) of these diseases, the use of human mesenchymalstem cells (hMSCs) is being proposed as an alternative to treat these patients. For instance, a study conducted by Joly et al. (8) reported an increase in the mortality rate and the occurrence of severe adverse effects such as sepsis and diabetes mellitus requiring insulin in patients with extensive bullous pemphigoid treated with 1 mg of prednisone per kilogram per day, compared to patients treated with only topical corticosteroids. In addition, despite being effective in the treatment of pemphigus (9, 10), the combination of rituximab and prednisone is associated with the occurrence of many adverse events such as diabetes, endocrine disorders, myopathy and bone disorders, which complicates the treatment of this disease (10). Other autoimmune diseases, such as epidermolysis bullosa acquisita are notoriously difficult to treat by the conventional treatment, as demonstrated in a study conducted by Kim et al. (11). This emphasizes the need for the elaboration of alternative therapies. In this regard, the use of human mesenchymal stem cells (hMSCs) has been studied as an alternative for the treatment of immune-related diseases due their intrinsic immunomodulatory properties.

Mesenchymal stem cells are multipotent cells capable of self renewal and differentiation into several cell lines, including chondrocytes, adipocytes and osteoblasts (12, 13). Despite the fact that this type of stem cells isusually isolated from bone marrow (12), they can also be obtained from several neonatal and adult tissues, including dental pulp (14), umbilical cord (15), orbicularis oris muscle (16), and fat (17). In addition, some studies reported successful differentiation of pluripotent stem cells such as embryonic stem cells and induced pluripotent stem cells into mesenchymal-like cells (18, 19). The therapeutic properties of hMSCs have been attributed to the secretion of factors with paracrine effects (20). Notably, hMSCs have been shown to be capable of supporting the maturation and proliferation of hematopoietic cells, migrating to an area of tissue injury, recruiting tissue-specific progenitor cells (21) and regulating the immune response through the secretion immunomodulatory cytokines and microvesicles containing a variety of bioactive molecules such as enzymes, coding and non-coding RNAs and growth factors (22). Regarding their immunomodulatory potential, hMSCs, when exposed to a pro-inflammatory stimulus, secrete molecules that modulate both innate and adaptive responses (23). These molecules secreted acts, for instance, in the inhibition of thematuration of monocytes in antigen-presenting dendritic cells (24), by promoting a shift from M1 to M2 macrophages (25), by inhibiting the proliferation and activation of B and T lymphocytes (26) and by promoting the clonal expansion of regulatory T lymphocytes (27).

Positive results from pre-clinical trials (28) and the demonstration of immunomodulatory effects of mesenchymal stem cells in “*in vitro*” experiments (29) led to a rapid increase in interest for the therapeutic potential of the administration of these cells for the treatment of several immune-related diseases (20). As a consequence, it is currently possible to isolate hMSCs from a variety of tissues (12, 14–17), expand them in culture and administer them locally (30) or intravenously (31) for treatment of immune-related diseases (32).

### Objectives

Therefore, the main objective of this study is to present and summarize, through a systematic review of the literature, *in vivo* studies in which the efficacy of the administration of hMSCs for the treatment of immune-related diseases was evaluated.

## Methodology

An electronic customized search of scientific articles published between 1984 and December 2017 using PubMed/MEDLINE, Scopus and Web of Science databases was conducted. The keywords used in the selection process were “mesenchymal stem cell AND (immunomodulation OR immunomodulatory therapy).” From the initial search, 864 studies were retrieved as potentially relevant from PubMed/Medline, 1,702 studies were retrieved as potentially relevant from Scopus and 1,545 studies were retrieved as potentially relevant from Web of Science database. As a result, it was identified a total of 4,111 articles containing the keywords used in the selection process. The application of the inclusion and exclusion criteria for each article was conducted by two independent researchers (ALJ and CP) through the screening of titles and abstracts. The inclusion criteria used to select the manuscripts were: to be studies published in English, to use human mesenchymal stem cells; to present the mesenchymal stem cell source used in the studies and to have results in concern to the evaluation of the immune-related diseases treatment through the administration of hMSC in animal models of immune-related diseases and also when these cells were applied in humans clinical trials studies. Duplicate articles were excluded from the analysis. Furthermore, were excluded: articles written in other languages than English; review manuscripts; *in vitro* studies; studies in which stem cells were not used; studies that used only non-human MSCs; and studies that evaluated the potential of MSCs for the treatment of non-autoimmune diseases (excluding graft-versus-host disease). Disagreements between the two independent researchers (AJ and CP) were identified and resolved by discussion with a third reviewer (DB). After this, the selected articles were reviewed and classified according to the type of immune-related disease studied, the source of the hMSCs isolated, the *in vivo* experimental model chosen, the clinical effects observed after administration of hMSCs and the proposed mechanisms of action of the hMSCs administered.

## Results

**Figure 1:**
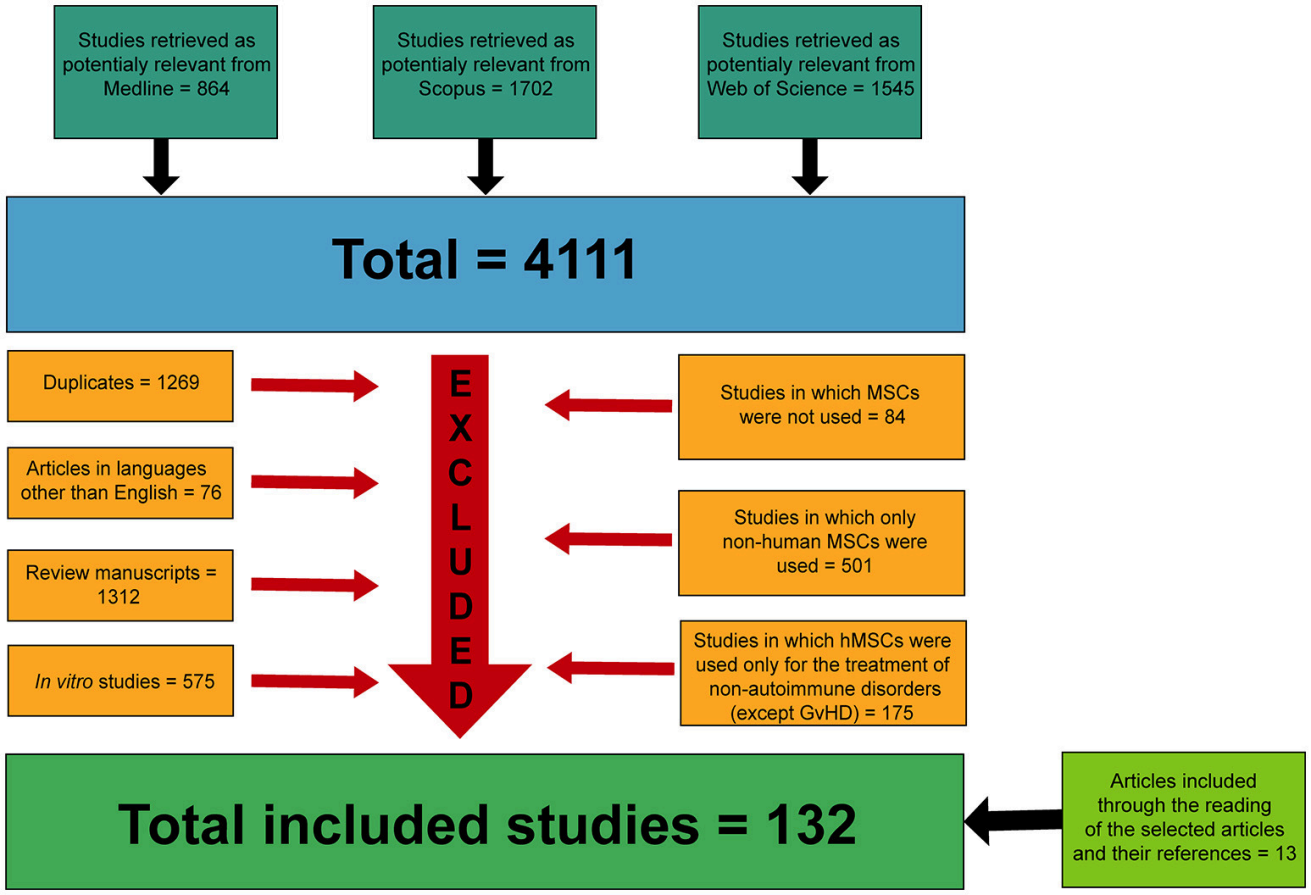
Flow diagram presenting the results of the literature search and the strategy used to select manuscripts in which hMSCs were used for the treatment of immune-related diseases.

The initial search resulted in 4,111 articles. Among them, 1,269 articles were excluded because they were duplicates, 76 articles written in languages other than English, 575 *in vitro* studies, 1,312 review manuscripts, 175 studies that evaluated the use of hMSCs for the treatment of non-immune-related diseases, 501 studies that used only non-human MSCs and 84 studies in which MSCs were not used were also removed from the analysis (Figure 1).

After the application of both exclusion and inclusion criteria, a total of 119 studies (33–151) were selected for analysis. Other 13 articles (152–164) were manually included for analysis in this systematic review after the reading of the articles previously selected and through the examination of their references. Therefore, a total of 132 manuscripts (33–164) formed the basis of this systematic review.

### The disease model

**Figure 2:**
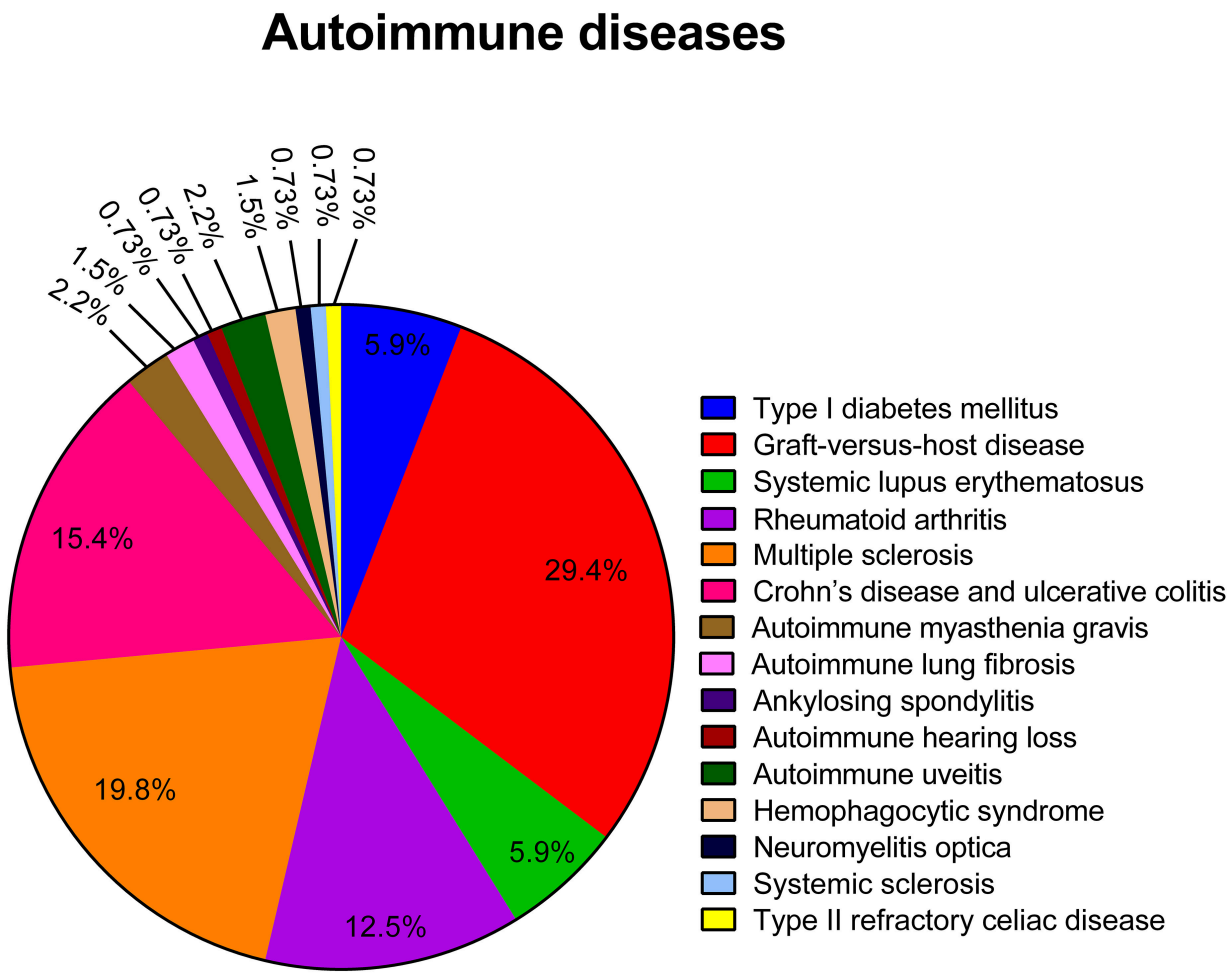
Representative graph of the different immune-related diseases for which hMSCs were used as therapeutic agents in the articles reviewed.

Medical complications that can occur following a hematopoietic stem cell transplantation (HSCT), such as graft-versus-host disease and hemophagocytic lymphohistiocytosis were treated with hMSCs in 40 (33–70–72) and two studies (52, 73), respectively. Chronic inflammatory disorders of the intestine such as Crohn's disease, ulcerative colitis and type II refractory celiac disease were treated with hMSCs in 19 (99–115, 155, 156), two (116, 117) and one (118) manuscript, respectively. Autoimmune joint diseases such as rheumatoid arthritis and ankylosing spondylitis were treated though the use of hMSCs in 17 (72, 86–97, 152, 153, 157, 158) and one study (98), respectively. The treatment of type I diabetes mellitus with hMSCs was conducted in a total of eight manuscripts (74–80, 164). hMSCs were additionally used for the treatment of systemic lupus erythematosus and systemic sclerosis in eight (81–84, 159–162) and one study (85), respectively. Autoimmune neurologic disorders such as multiple sclerosis, autoimmune myasthenia gravis and neuromyelitis optica were treated with hMSCs in 27 (102, 121–145, 154), three (146–148) and one study (149), respectively. Autoimmune visual and auditory disorders such and autoimmune uveitis and autoimmune hearing loss were treated with hMSCs in three (81, 150, 163) and one study (151), respectively. Finally, two studies (119, 120) applied hMSCs for the treatment of autoimmune-disease associated lung fibrosis. The use of hMSCs for the treatment of the immune-related diseases studied in the articles reviewed is graphically represented on Figure 2.

### The source of hMSCs

**Figure 3:**
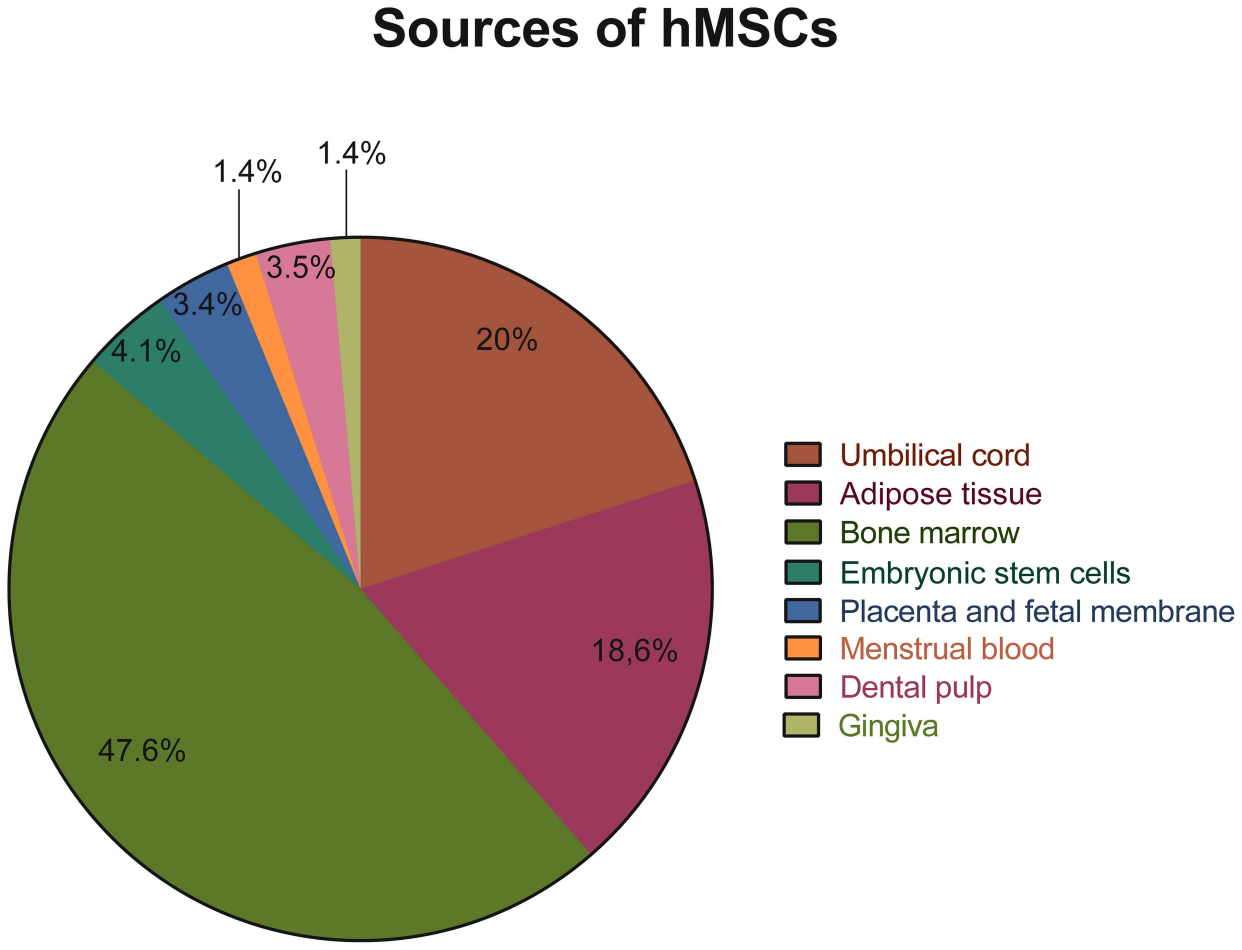
Representative graph of the different sources used for the isolation of hMSCs in the articles reviewed.

As expected, the bone marrow was chosen as the source of hMSCs in the majority of the articles analyzed. A total of 69 out of the 132 articles selected isolated hMSCs from the bone marrow (35, 37, 42–45, 47, 49–58, 60, 62–64, 66–73, 77, 79, 80, 92, 95, 98, 99, 102, 107, 108, 110, 111, 115–118, 122, 123, 125–128, 131, 132, 133, 134, 135, 137, 138, 143, 146, 148–150, 154–158, 161). In addition, other common sources of hMSCs included the umbilical cord blood or stroma [29 articles (36, 38, 39, 41, 46, 61, 65, 75, 76, 78, 82, 85, 89, 91, 93, 95, 97, 100, 101, 103, 107, 113, 119, 124, 128, 142, 144, 155, 158)] and the adipose tissue [27 articles (40, 59, 74, 83, 86, 87, 94, 96, 100, 103, 104, 106, 110, 120, 121, 128, 130, 136, 140, 141, 145, 151–153, 156, 158, 159)]. On the other hand, the dental pulp was chosen as the source of hMSCs in only five articles (33, 84, 114, 147, 162), in two articles hMSCs were isolated from the gingiva (90, 99), in two articles hMSCs were obtained from the menstrual blood (72, 105) and in five articles (34, 48, 88, 129, 139) hMSCs were isolated from extra embryonic membranes such as the placenta and fetal membrane. Finally, in six studies (81, 102, 103, 137, 157, 160) hMSCs were obtained from the directed differentiation of embryonic stem cells. The sources of hMSCs used for the isolation of hMSCs in the articles reviewed are graphically represented on Figure 3.

### The experimental model

**Figure 4:**
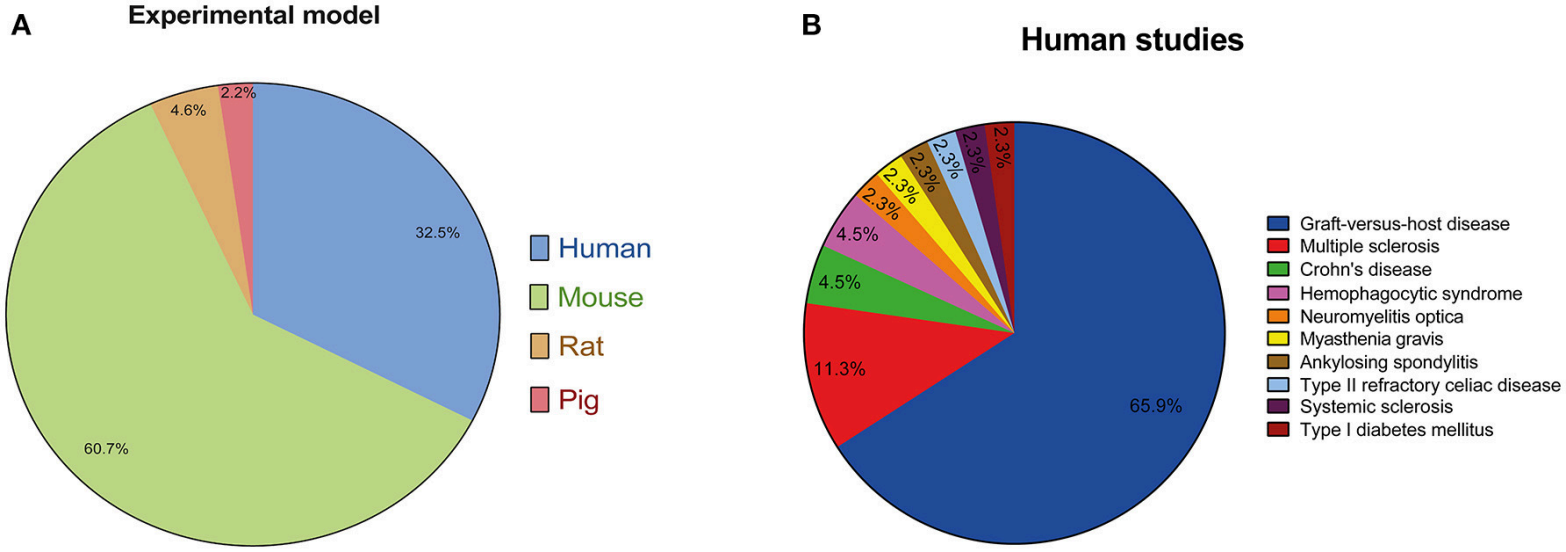
**(A)** Representative graph of the different experimental models employed to assess the therapeutic potential of the use of hMSCs for the treatment of immune-related diseases in the articles reviewed. **(B)** Representative graph of the different articles in which hMSCs were used for the treatment of immune-related diseases in humans studies. It is important to notice that, in the majority of studies, hMSCs were administered for the treatment of graft-versus-host disease following HSCT.

Fourty-three (42–47, 49–71, 73, 78, 85, 98, 116, 117, 118, 125, 131–135, 148, 149) out of the 132 studies (33, 35–41, 48, 72, 74–77, 79–84, 86–97, 99–115, 119–124, 126–130, 133, 136–147, 150–164) selected were conducted in humans, and, in 89 manuscripts (33–164>, 91, 92, 93, 94, 95, 96, 97, 98, 99, 100, 101, 102, 103, 104, 105, 106, 107, 108, 109, 110, 111, 112, 113, 114, 115, 116–118, 119, 120, 121, 122, 123, 124, 125, 126, 127, 128, 129, 130, 131, 132, 133, 134, 135, 136, 137, 138, 139, 140, 141, 142, 143–147, 149, 150–164) selected were conducted in humans, and, in 89 manuscripts (33–164), animal models were applied for the study of the therapeutic effects of the administration of hMSCs for the treatment of immune-related diseases. Among the articles that used animal models, 80 used mice (33–41, 48, 72, 74–77, 79–84, 86, 87, 89–94, 96, 99–107, 109, 110, 113–115, 119–129, 133, 136–139, 141, 143–147, 150–164), six used rats (88, 95, 97, 130, 140, 142) and three used pigs (108, 111, 112) as the experimental model. The use of these different experimental models in the articles reviewed is graphically represented on Figure 4. It is important to notice that, in 29 (42–47, 49–71) out of the fourth-two studies conducted in humans, hMSCs were administered for the treatment of graft-versus-host disease following HSCT. In addition, in two human studies (52, 73), hMSCs were used for the treatment of hemophagocytic syndrome, in five studies (125, 131, 132, 134, 135) they were used for the treatment of multiple sclerosis and in two studies (116, 117) they were used for the treatment of Crohn's disease. The treatment of neuromyelitis optica (149), myasthenia gravis (148), ankylosing spondylitis (98), type II refractory celiac disease (118), systemic sclerosis (85) and type I diabetes mellitus (78) was conducted in humans in only one article each. The use of hMSCs for the treatment of immune-related diseases in human studies is graphically represented on Figure 4.

### Outcomes

All the articles selected were analyzed individually and categorized according to the immune-related disease treated with hMSCs, the source of the hMSCs used and the experimental model employed. Furthermore, the clinical effects and the mechanism of action of the hMSCs administered for the treatment of the immune-related diseases studied by the articles reviewed were also analyzed individually.

#### Treatment of graft-versus-host disease and hemophagocytic syndrome

Graft-versus-host disease (GvHD) is a systemic syndrome that can occur following a hematopoietic stem cell transplantation (HSCT) (165). This disease results from the activation of donor-derived T lymphocytes by histocompatibility antigens from host tissues and leads to the attack of the host's body cells by these activated donor-derived T cells (166). The therapeutic potential of the administration of hMSCs for the treatment of graft-versus-host disease was investigated in 40 (33–72) out of the 132 articles (33–164) analyzed. Among them, 29 were conducted in humans (42–47, 49–71) and 11 used mice as the experimental model (33, 34, 35–41, 48, 72). Regarding the source of the hMSCs used, in 28 studies hMSCs were isolated from the bone marrow (35, 37, 42–47, 49–71, 72), in seven studies (36, 38, 39, 41, 46, 61, 65) they were isolated from the umbilical cord and in two (40, 59) from the adipose tissue. The menstrual blood (72), dental pulp (33), placenta (48) and fetal membrane (34) was used as a source of hMSCs in only one study each.

Based on timing of onset after HSCT and according to the clinical manifestations observed, GvHD can be classified as acute or chronic. Acute GvHD usually affects the skin, liver and gastrointestinal tract of the patients affected by the disease (167). On the other hand, chronic GvHD can affect any organ (168). Clinical effects commonly observed after the administration of hMSCs included an increase in the survival rates (35, 36, 39–41, 44, 45, 48, 49, 51, 56–62, 65–68, 71, 72), a decrease in the severity of the symptoms of the disease (33–36, 38–46, 52–59, 61–64, 66–68, 71) and a reduction in the incidence of acute and chronic GvHD (51, 53, 54, 65) in patients submitted to hematopoietic stem cell transplantation. Among the studies conducted in mice, the cumulative survival rate, the clinical score of the disease and the rate of change in body weight were the outcomes used by most animal studies selected in this systematic review to assess the potential of hMSCs administration for the treatment of GvHD. Among the human clinical trials selected, the primary endpoints used by most studies to assess the effectiveness of the treatment with hMSCs were: the overall survival rate; the disease-free survival rate; the progression-free survival rate; the non-relapse mortality rate; and the relapse incidence. We propose that both the disease-free survival rate and the progression-free survival rate are the most appropriate endpoints for future clinical trials when evaluating the effectiveness of hMSCs administration for the treatment of GvHD. While the disease-free survival rate should be used for patients in remission at time of HSCT, the progression-free survival rate should be used for patients that were not in remission at time of HSCT. These two primary endpoints were chosen because they are more likely to not be affected by bias, are detected earlier than the overall survival rate, include all clinically important events evaluated, and are more likely to reflect the real benefits of the treatment. Furthermore, additional endpoints such as the cumulative incidence of grade II-IV acute GVHD, the cumulative incidence of grade III-IV acute GVHD, and the cumulative incidence of moderate or severe chronic GVHD can also be used in combination with the primary endpoints selected in order to answer to other important questions about the benefits of the treatment.

In addition to the amelioration of symptoms and decrease in mortality, many studies observed a significant decrease in the pathology of the gut (35, 36, 39, 41, 48, 56, 59, 64, 67, 72), liver (35, 36, 39, 41, 48, 56, 59, 67), skin (36, 48, 56, 59, 64, 67), lungs (41, 65), and kidneys (41) of patients treated with hMSCs. In conjunction with this reduction in the pathological state, some studies also described a decrease in the serum concentration of local tissue damage biomarkers such as the markers of epithelial damage elafin (71), ccK18 (50), and K18 (50) and the markers of gastrointestinal damage sCK18f (66), Reg3α (71), and CK18 (66, 71). On the other hand, adverse effects observed after the administration of hMSCs included an increase in the rates of pneumonia (47) and infection-related death (49). Additionally, the occurrence of fatal embolism was also found to be significantly associated with the administration of hMSCs in one study (33) and, in another study (70), the reconstitution of both T and B cell function was found to be worsened after hMSCs administration. However, a study conducted by Guo et al. (43) showed that CD3^−^CD16^+^56^+^(NK) and CD3^+^CD16^+^56^+^(NKT) cells and CD3^+^CD8^+^ T cells were upregulated in 1–3 months after transplantation when hMSCs were administered, showing that the administration of hMSCs may be important in reducing leukemia relapse after HSCT.

While the development of acute GvHD is related to the activation of alloreactive T lymphocytes of the graft, the development of chronic GvHD involves both alloreactive and autoreactive mechanisms (169). The immune response of acute GvHD occurs in two phases, one afferent and one efferent. In the afferent phase, CD4^+^ and CD8^+^ T cells react to the host's class I and II alloantigens present on the surface of antigen-presenting cells (APCs) (166). This phase starts when the conditioning regimen initiates an immune response due to the damage to host tissues, such as the intestinal mucosa and liver, which results in the induction of cytokine secretion, especially IL-1 and tumor necrosis TNF-α (170). After HSCT, donor's T cells are stimulated by the IL-1 and by the costimulatory signals present, producing IL-2. Under the influence of IL-2, CD4+ and CD8+ T cells clonally expand and differentiate into effector cells, which induce the graft response against the host (170). These effector cells are activated by costimulatory molecules and proinflammatory cytokines such as IFN-γ and IL-12, giving rise to T helper 1 (Th1) effector cells, which direct even more the graft response against the host (171). In the efferent phase of GvHD, activated T cells secrete a storm of cytokines such as IL-2, IL-4, IL-3, and IFN-γ. These mediators recruit and activate effector cells, including additional lymphocytes, macrophages, and natural killer (NK) cells, which attack both donor and host tissues (170). The mechanisms of action of the hMSCs administered included effects in the proliferation and differentiation of immune cells and changes in the expression pattern of growth factors, cytokines, enzymes, prostaglandins and surface receptors and ligands. While some studies reported an increase in the levels of growth factors such as HGF (34, 72), IGF-1 (34), VEGF (34, 72), bFGF (34, 71), TGF-β (39, 41), activin A (72), and NGF (71), others demonstrated an increase in the levels of the prostaglandin E2 (34, 41, 72) (PGE2) and the enzymes IDO (39, 72), Cox-2 (72), and granzyme B (71). Generally, an increase in levels of immunomodulatory cytokines such as IL-10 (36, 58, 71) and IL-23 (71) and a decrease in the levels of pro-inflammatory cytokines such as TNF-α (35, 36, 55, 64), IFN-γ (36, 41, 55, 64), IL-1β (64), IL-2 (36), IL-8 (71), CCL2 (71), CXCL9 (71), and CXCL10 (71) following treatment with hMSCs was observed by most studies. In addition, some studies reported an increase in the serum levels of IL-6 (50, 72), a cytokine with both pro-inflammatory and anti-inflammatory properties, after administration of hMSCs while another study reported an increase in the levels of GM-CSF (71), a cytokine usually employed to stimulate the production of leukocytes in order to prevent neutropenia after chemotherapy. A change in the expression of cell surface receptors and ligands following the administration of hMSCs for the treatment of GvHD was also demonstrated by some studies. For instance, a decrease in the expression of PPAR-γ (51), IL-2 (49, 66, 71), and TNF-α (66, 71) receptors and in the CD40 ligand (71) (CD40L) was observed in some articles while an increase in the expression of the protein receptor CTLA-4 (58) and NRP-1 (72) and in the PD-ligand 1 (72) (PD-L1) and CCR2 (71) ligand (CCL7) was demonstrated by other studies.

Many of the studies selected reported an inhibitory effect in the proliferation of both B (55) and T (35, 36, 69, 72) cells after treatment with hMSCs. Some of the studies selected described a decrease in the proliferation of both CD8^+^ (50, 72) and CD4^+^ (38, 39, 50, 59) T cells after treatment of GvHD with hMSCs. However, an increase in the CD4^+^/CD8^+^ T cell ratio was also commonly observed (36, 50, 53). Specifically, some studies reported that the administration of hMSCs suppressed the clonal expansion of CD4^+^IFN-γ^+^ Th1 (40, 72) and CD4^+^IL-17^+^ Th17 (40, 50, 59, 72) cells while exhibiting an opposite effect on CD4^+^IL-4^+^ Th2 (40) and CD4^+^CD25^+^Foxp3^+^ Treg (40, 50, 53, 55, 58, 59, 65, 72) cells. In addition, a study conducted by Weng et al. (42) demonstrated that the administration of hMSCs stimulated the generation of CD8^+^CD28^−^ T cells, which may regulate the balance between Th1 and Th2 responses. Regarding the effects of hMSCs administration in the proliferation and differentiation of B cells, a study conducted by Zhang et al. (55) demonstrated that the treatment with hMSCs inhibited the proliferation of CD19^+^ B cells and increased the proportion of CD5^+^IL-10^+^Breg cells within the CD19^+^ B cell population. On the other hand, Gao et al. (65) reported an increase in the proliferation of CD27^+^ memory B lymphocytes after the administration of hMSCs. Effects in the proliferation of NK cells following the administration of hMSCs were also observed in some studies. For instance, a study conducted by Jitschin et al. (50) found that the proportion of activated CD56^bright^ NK-cells was significantly lower in patients treated with hMSCs compared to untreated patients while Gao et al. (65) described a decrease in the total number of NK cells following hMSCs administration. Some studies also reported effects in the infiltration of immune cells in organs typically affected by GvHD after treatment with hMSCs. For instance, Gregoire-Gauthier et al. (38) described that the infiltration of CD4^+^ T helper cells was found to be decreased in the liver and increased in spleen of acute GvHD mice after hMSCs administration. On the other hand, in a study conducted by Luz-Crawford et al. (72), infiltration of CD8^+^ cytotoxic T cells in spleen was found to be either decreased or increased after hMSCs administration, depending on the source of hMSCs used. Furthermore, according to Girdlestone et al. (61), administration of hMSCs previously treated with rapamycin significantly inhibited the infiltration of CD45^+^ cells in the spleen of acute GvHD mice.

**Figure 5:**
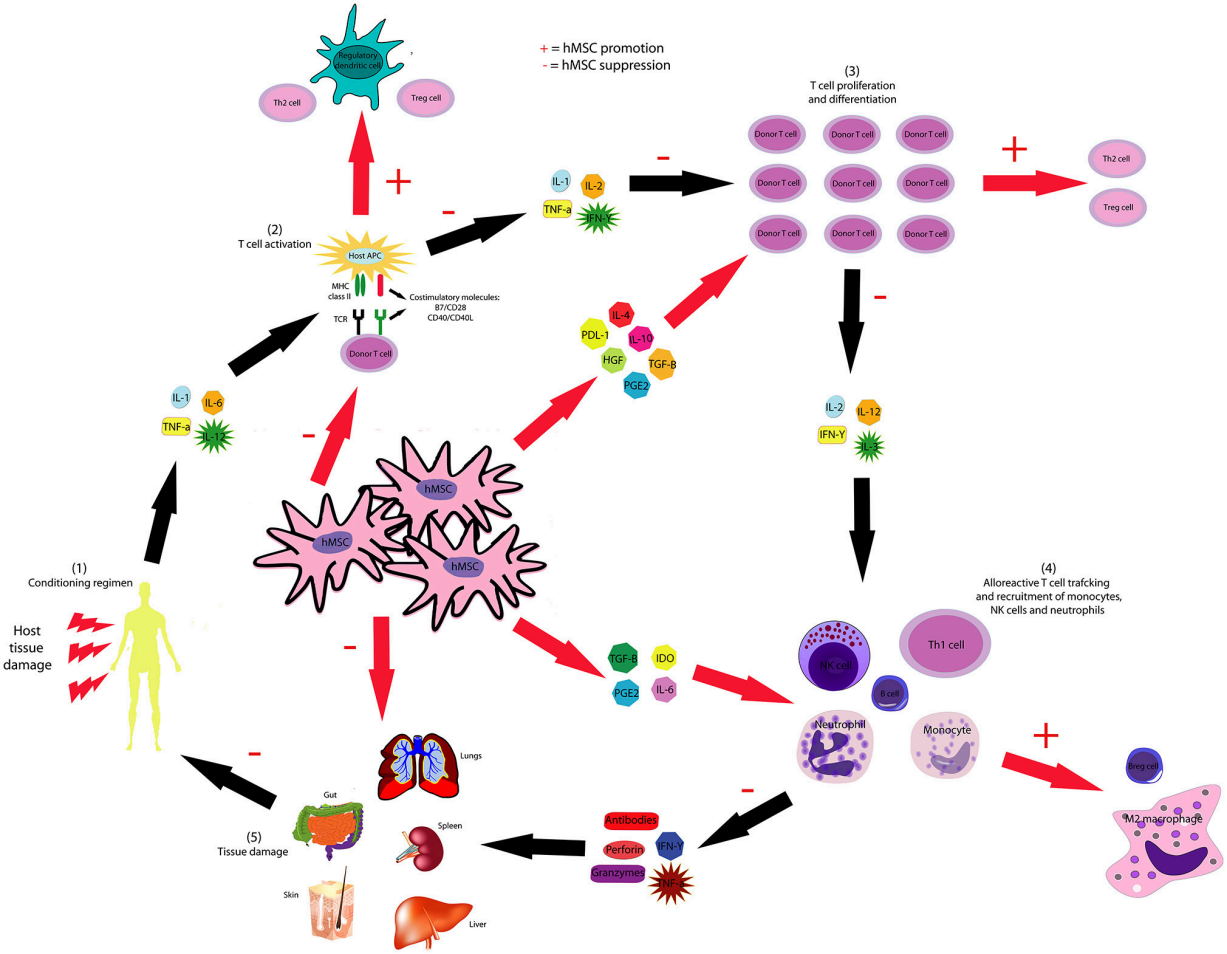
hMSCs inhibit the pathological course of GvHD through several mechanisms. hMSC-produced IL-4, IL-10, HGF, PGE2, PDL-1, and TGF-β inhibit the proliferation and activation of T and B cells and stimulate the generation of Breg, Treg, and Th2 lymphocytes. hMSCs inhibit the activation of dendritic cells and stimulate the generation of regulatory dendritic cells. hMSC-produced IL-6, IDO, PGE2, and TGF-β suppresses neutrophil respiratory burst, NK cell activation and macrophage polarization to M1, though favors M2 polarization.

It is possible that the secretion of immunomodulatory cytokines and growth factors by hMSCs strongly influences this inhibitory effect observed in the proliferation of B and T cells after hMSCs administration. For instance, secretion of IL-4, IL-10, TGF-β, HGF, PGE2, and PDL-1 may act on donor T cells and inhibit their activation, proliferation and differentiation into Th1 cells and stimulates their differentiation into Th2 lymphocytes and antiinflammatory Treg lymphocytes. As a consequence, the secretion of pro-inflammatory cytokines such as IL-2, IL-3, IL-12, and IFN-γ by donor T cells is also inhibited, which decreases the trafficking of reactive T cells, the recruitment of B cells, monocytes, neutrophils and NK cells and the secretion of granzymes, perforin, IFN-γ, TNF-α and antibodies by these cells. In addition, secretion of immunomodulatory cytokines and growth factors by hMSCs may act on the cells recruited by donor T cells, inhibiting their proliferation and stimulating their differentiation into immunomodulatory cells such regulatory dendritic cells, Breg cells and M2 macrophages. Therefore, the damage to organs such as lungs, spleen, gut, skin and liver would also be decreased. As a result of this reduction in the damage to host tissues, the induction of cytokine secretion in these tissues is also inhibited, further inhibiting the occurrence of the pathological process of GvHD. The mechanisms proposed by this systematic review concerning the inhibition in the progression of the pathological process of GvHD mediated by hMSCs are represented in Figure 5.

Hemophagocytic syndrome is an autoimmune disease characterized by the activation and proliferation of macrophages, T CD8^+^ lymphocytes and NK cells in the bone marrow and in other endothelial reticular systems (172), leading to the phagocytosis of erythrocytes, leukocytes, platelets, and their precursors and to the exacerbated production of several inflammatory cytokines, including IL-1β, IL-2, IL-6, TNF-α, and IFN-γ (173). The hemophagocytic syndrome may be primary when triggered by genetic factors or secondary when occurs due to infections, neoplasms, rheumatic diseases, HSCT or other autoimmune disorders. The clinical manifestations of the hemophagocytic syndrome include hyperferritinemia, fever, hepatosplenomegaly and cytopenias (174). In this systematic review, hMSCs were used for the treatment of the hemophagocytic syndrome in only two studies (52, 73); both of which had the bone marrow as the source of hMSCs and were conducted in humans. Both the studies selected were single case reports and used the platelet, leukocyte and reticulocyte number, the amount of hemophagocytosis of erythroblasts and myeloid cells and the serum levels of ferritin and lactate dehydrogenase as primary study endpoints. The serum levels of pro-inflammatory and immunomodulatory cytokines such as IL-1β, IL-6, IL-8, IL-10, IL-17, and IL-15 were used as secondary study endpoints as they provide a strong evidence of the efficacy of the hMSCs administration inmodulating autoimmunity. Due to the fact that the hemophagocytic syndrome is a universally fatal disease if untreated, we recommend the use of a primary endpoint that is able to reflect a change in lethality following hMSCs administration, such as the cumulative survival rate. Other outcomes such as neutropenia, occurrence of relapses and serum levels of cytokines should be used in conjunction with the primary endpoint to correctly assess the potential of hMSCs administration for the treatment of hemophagocytic syndrome. After hMSCs administration, a decrease in the severity of the disease was observed in both studies following the administration of hMSCs. Mougiakakos et al. (73) described that this decrease in the severity of the disease was accompanied by a reduction in the serum levels of lactate dehydrogenase, ferritin and triglycerides. This study also described an increase in the levels of the immunomodulatory cytokine IL-10 and a decrease in the levels of the inflammatory cytokines IL-8, IL-6, IL-15, IL-17, and TNF-α after treatment with hMSCs. Table 1 summarizes the methodology employed and the results obtained in the studies selected in this systematic review regarding the effects of the administration of hMSCs for the treatment of GvHD and hemophagocytic syndrome.

**Table 1:** List of *in vivo* studies in which the therapeutic potential of the administration of hMSCs for the treatment of GvHD and hemophagocytic syndrome was evaluated and the results obtained.

**References**	**Autoimmune disease**	**Source of hMSC**	**Variables**	**Experimental model**	**Clinical and laboratory effects**	**Proposed mechanisms for the *in vivo* action of MSCs**
(33)	GVHD	Dental pulp	Administration of MSCs alone Administration of MSCs transduced with immunosuppressive genes	Mice	↓Clinical score ↑Fatal embolism Effectiveness MSC transduced with immunosuppressive genes = effectiveness of MSCs alone	↓Mouse splenocyte proliferation
(34)	GVHD	Fetal membrane	Administration of MSCs from amnion membrane	Mice	↓Weight loss Effectiveness MSC from amnion membrane > effectiveness of MSCs from from chorion membrane	↑HGF secretion ↑IGF-1 secretion ↑VEGF secretion ↑bFGF secretion ↑PGE2 secretion ↓T-cell proliferation
			Administration of MSCs from chorion membrane		None	
(35)	GVHD	Bone marrow	Infusion of MSCs on day 0	Mice	None	↓Donor T cell proliferation ↓TNF-α
			Infusion of MSCs on day 7		↓Weight loss ↑Survival rates ↓Acute GvHD score ↓Gut and liver pathology	
			Stimulation of MSCs with IFN-γ for 48 h prior to administration on day 0		↓Weight loss ↑Survival rates ↓Clinical score ↓Gut and liver pathology	
(36)	GVHD	Umbilical cord stroma	None	Mice	↓Clinical score ↓Weight loss ↑Survival rates ↓Gut, skin and liver pathology	↓CD3^+^CD8^+^ T cells ↑CD4^+^/CD8^+^ ratio ↓TNF-α ↓IL-2 ↓IFN- γ ↑IL-10
(37)	GVHD	Bone marrow	None	Mice	None	↓T-cell proliferation ↓IFN- γ secretion
(38)	GVHD	Umbilical cord blood	Presence of radiation-induced damage	Mice	↓Weight loss	↓Human/mice CD45^+^ cells ratio ↑Human CD45^+^ cells total number ↓Human CD3^+^ cells in the liver ↓Human CD4^+^ cells in the liver
			Absence of radiation-induced damage		↓Clinical score ↑Survival rates	↓Human/mice CD45^+^ cells ratio ↓Human CD45^+^ cells total number ↓Human CD3^+^ cells in the liver ↓Human CD4^+^ cells in the liver
(39)	GVHD	Umbilical cord stroma	Stimulation of MSCs with IFN-γ for 24 h	Mice	Not assessed	↓CD4^+^ T-cell proliferation ↑IDO gene expression ↑TGF-β gene expression
			Absence of stimulation of MSCs with IFN-γ for 24 h		↓Clinical score ↓Weight loss ↑Survival rates ↓Gut and liver pathology	↑TGF-β gene expression
(40)	GVHD	Adipose tissue	Absence of stimulation of MSCs with rapamycin prior to administration Stimulation of MSCs with rapamycin prior to administration	Mice	↓Clinical score ↑Survival rates ↓Weight loss Effectiveness MSCs + rapamycin > effectiveness MSCs alone	↓CD4^+^IFN-γ^+^ Th1 cells ↓CD4^+^IL-17^+^ Th17 cells ↑CD4^+^IL-4^+^ Th2 cells ↑CD4^+^CD25^+^Foxp3^+^ Treg cells
(41)	GVHD	Umbilical cord blood	Prevention study for GVHDTreatment study for GVHD	Mice	↑Survival rates ↓Weight loss ↓Clinical score ↓Kidney, lungs, liver and gut pathology Multiple MSCs administrations > single MSCs administration at day 0.	↑PGE2 ↑TGF-β1 ↓IFN-γ
(48)	GVHD	Placenta	None	Mice	↑Survival rates ↓Weight loss ↓Gut, skin and liver pathology	None
(59)	GVHD	Adipose tissue	Co-infusion of MSCs and Tregs Administration of MSCs alone	Mice	↑Survival rates ↓Weight loss ↓Clinical score ↓Gut, skin and liver pathology Effectiveness MSCs + Tregs > effectiveness MSCs alone	↓CD3^+^CD4^+^ T-cells ↓Th17 cells ↑Foxp3^+^ Tregs cells
(61)	GVHD	Umbilical cord blood	Infusion of MSCs previously treated with rapamycinAdministration of MSCs aloneMSCs from pooled bone marrow mononuclear cells of eight “3rd-party” donors (MSCs end-products)	Mice	↑Survival rates ↓Weight loss Effectiveness MSCs + rapamycin > effectiveness MSCs alone	↓Infiltration of human CD45^+^ cells in the spleen (MSCs pre-treated with rapamycin)
(72)	GVHD	Bone marrow Menstrual blood	None	Mice	↑Survival rates ↓Weight loss ↓Gut and liver pathology Effectiveness MSCs from menstrual blood > effectiveness MSCs from bone marrow	↓CD8^+^IFN-γ^+^ cells ↓CD4^+^IFN-γ^+^ Th1 cells ↓Th17 cells ↑CD4^+^IL4^+^IL10^+^ T cells ↑Tregs cells ↑IDO ↑PD-L1 ↑PGE2 ↑Activin A ↑Cox-2 ↑IFN-γ (menstrual blood MSCs) ↓Foxp3^+^ expression in splenocytes (menstrual blood MSCs) ↑IL-6 expression ↑NRP-1 expression ↑HGF expression in the liver (menstrual blood MSCs) ↑VEGF expression in the liver (menstrual blood MSCs)
						↑CXCR4^+^ cells (in the menstrual blood MSCs population) ↓Human CD45^+^ cells in spleen (bone marrow MSCs) ↓Human CD45^+^CD8^+^ T cells in spleen (bone marrow MSCs) ↑ Human CD45^+^CD8^+^ T cells in spleen (menstrual blood MSCs) ↑ Human CD45^+^CD4^+^ T cells in spleen (menstrual blood and bone marrow MSCs)
(42)	GVHD	Bone marrow	None	Humans	↓Dry eye symptoms ↓GvHD clinical score	↑CD8^+^CD28^−^ T cells ↑IL-2 ↑IFN-γ ↓IL-10 ↓IL-4
(43)	GVHD	Bone marrow	None	Humans	↑Donor engraftment ↓Clinical score ↓Leukemia relapse	↑CD3^+^CD8^+^ T cell reconstitution ↑CD3^−^CD(16+56)^+^ T cells reconstitution ↑CD3^+^CD(16+56)^+^ T cells reconstitution
(44)	GVHD	Bone marrow	None	Humans	↑Survival rates ↓Clinical score	None
(45)	GVHD	Bone marrow	None	Humans	↑Survival rates ↓Clinical score	↓CD3^+^/CD4^+^ T cells ratio
(46)	GVHD	Umbilical cord blood	None	Humans	↑Donor engraftment ↓Clinical score	None
(47)	GVHD	Bone marrow	None	Humans	↑Pneumonia-related death	None
(49)	GVHD	Bone marrow	MSCs at first or second passage	Humans	↑Survival rates ↑Infection-related death Effectiveness MSCs at first or second passage > effectiveness MSCs at third or fourth passage	↓IL-2 receptor in the serum
			MSCs at third or fourth passage			
(50)	GVHD	Bone marrow	None	Humans	↓ccK18 ↓K18	↑IL-2 ↑IL-6 ↓IFN-γ/IL-4 ratio ↑CD4^+^/CD8^+^ ratio ↓HLA-DR^+^CD4^+^ T-cells ↓HLA-DR^+^CD8^+^ T-cells ↑CD4^+^CD25^med−hi^ CD127^lo^FOXP3^+^ Treg-cells ↓Th17 cells ↑Treg/Th17 ratio ↓CD56^bright^ NK-cells
(51)	GVHD	Bone marrow	None	Humans	↑Acute GVHD disease prophylaxis ↑Survival rates	↑FGF receptor gene ↓PPAR-γ gene ↓IGF-1 gene
(52)	GVHD	Bone marrow	None	Humans	↓Clinical score	None
(53)	GVHD	Bone marrow	None	Humans	↓Clinical score ↓Chronic GVHD incidence	↑CD4^+^/CD8^+^ ratio ↑CD4^+^CD25^+^Foxp3^+^ Tregs cells ↑T cell reconstitution (↑sjTRECs)
(54)	GVHD	Bone marrow	None	Humans	↓Acute GVHD incidence ↓Clinical score	None
(55)	GVHD	Bone marrow	None	Humans	↓Nephrotic syndrome symptoms	↓IFN-γ ↓TNF-α ↓CD19^+^ B cell ↑Bregs cells ↑Tregs cells
(56)	GVHD	Bone marrow	None	Humans	↓Clinical score ↑Survival rates ↓Gut, skin and liver pathology	None
(57)	GVHD	Bone marrow	None	Humans	↓Clinical score ↑Survival rates	None
(58)	GVHD	Bone marrow	None	Humans	↑Survival rates	↑IL-2 receptor lymphocyte gene expression ↑IFN- γ lymphocyte gene expression ↑FoxP3 lymphocyte gene expression ↑CTLA-4 lymphocyte gene expression ↑IL-10 lymphocyte gene expression ↑Foxp3^+^ Tregs cells
(60)	GVHD	Bone marrow	None	Humans	↑Survival rates	↑Anti-fetal calf serum antibodies ↓Alloantibodies
(62)	GVHD	Bone marrow	None	Humans	↑Survival rates ↓Clinical score	None
(63)	GVHD	Bone marrow	None	Humans	↓Clinical score ↓Bilirubin concentration	None
(64)	GVHD	Bone marrow	None	Humans	↓Clinical score ↓Skin and mucosal pathology	↓IL-1β ↓IFN-γ ↓TNF-α
(65)	GVHD	Umbilical cord stroma	None	Humans	↓Chronic GVHD incidence ↓Lung pathology ↑Survival rates	↑Th1/Th2 cells ratio ↑Treg cells ↑CD27^+^ memory B lymphocytes ↓NK cells
(66)	GVHD	Bone marrow	None	Humans	↑Survival rates ↓Gastrointestinal acute GVHD symptoms ↓Gastrointestinal pathology ↓CK18 ↓sCK18F ↓sCK18F/CK18 ratio	↓TNF-α receptor ↓IL-2 receptor
(67)	GVHD	Bone marrow	None	Humans	↑Survival rates ↓Clinical score ↓Gut, skin and liver pathology	None
(68)	GVHD	Bone marrow	MSCs from single donorsMSCs from pooled bone marrow mononuclear cells of eight “3rd-party” donors (MSCs end-products)	Humans	↑Survival rates ↓Clinical score Effectiveness MSCs end-products > effectiveness MSCs from single donors	None
(69)	GVHD	Bone marrow	None	Humans	↑Donor engraftment	↓Donor T-cell proliferation
(70)	GVHD	Bone marrow	None	Humans	↓Survival rates	↓T cell reconstitution (↓TRECs) ↓B cell reconstitution (↓IgM) ↓IgG
(71)	GVHD	Bone marrow	None	Humans	↑Survival rates ↓Clinical score ↓CK18	↓TNF-α receptor ↓Elafin ↓IL-2 receptor ↓Reg3α ↓HGF ↓IL-8 ↓CCL2 ↓CD40L ↓CXCL9 ↓CXCL10 ↑NGF ↑IL-10 ↑IL-12 ↑IFN-γ ↑IL-15 ↑CCL7 ↑bFGF ↑GM-CSF ↑TNF-α ↑IL-23 ↑Granzyme B
(52)	Hemophagocytic syndrome	Bone marrow	None	Humans	↓Clinical score	Not assessed
(73)	Hemophagocytic syndrome	Bone marrow	None	Humans	↓Disease severity ↓Serum ferritin ↓Serum triglycerides ↓Serum lactate dehydrogenase	↑IL-10 ↓IL-8 ↓IL-6 ↓IL-15 ↓IL-17 ↓TNF-α

*Both the methodology employed and the results obtained by each article are represented in this table. HGF, hepatocyte growth factor; IGF-1, insulin like growth factor 1; VEGF, vascular endothelial growth factor; bFGF, basic fibroblast growth factor; PGE2, prostaglandin E2; TNF-α, tumor necrosis factoralpha; IL-2, interleukin-2; IL-4, interleukin-4; IL-1β, interleukin-1 beta; IL-6, interleukin-6; IL-8, interleukin-8; IL-10, interleukin-10; IL-12, interleukin-12; IL-15, interleukin-15; IL-23, interleukin-23; IDO, indoleamine-pyrrole 2,3-dioxygenase; TGF-β, transforming growth factor beta; NGF, nerve growth factor; NK cells, natural killer cells; Th1 cells, type 1 T helper cells; Th2 cells, type 2 T helper cells; Th17 cells, type 17 T helper cells; Treg cells, regulatory T cells; Bregs cells, regulatory B cells; ccK18, caspase-cleaved cytokeratin 18; K18, keratin 18; CK18, cytoskeletal keratin 18; sCK18F, soluble cytokeratin 18 fragments; PPAR-γ, peroxisome proliferator-activated receptor; sjTRECs, signal joint T-cell receptor excision circles; TRECs, T-cell receptor excision circles; CCL2, C-C motif chemokine ligand 2; CCL7, C-C motif chemokine ligand 7; CXCR4, C-X-C chemokine receptor type 4; CXCL9, C-X-C motif chemokine ligand 9; CXCL10, C-X-C motif chemokine ligand 10; CTLA-4, cytotoxic T-lymphocyte-associated protein 4; IgM, immunoglobulin M; IgG, immunoglobulin G; Reg3α, regenerating islet derived protein 3 alpha; GM-CSF, granulocyte-macrophage colony-stimulating factor; PDL-1, programmed death-ligand 1; Cox-2, cyclooxygenase-2; NRP-1, neuropilin-1*.

#### Treatment of type I diabetes mellitus

Type 1 diabetes mellitus is a chronic metabolic disease characterized by an insulin deficiency caused by the cellular-mediated autoimmune destruction of the β-cells of the pancreas (175). The process of destruction of pancreatic β cells, called insulitis, is a consequence of an immunological attack mediated by lymphocytes, macrophages and NK cells and leads to a permanent hyperglycemia and the need for exogenous insulin replacement (176). CD8^+^ T lymphocytes are the predominant type of immune cell responsible for the insulitis process, but the presence of CD4^+^ T lymphocytes and B lymphocytes can also be detected in the lymphocytic infiltrate in pancreatic islets (177). Furthermore, the cytokine secretion profile during the development of type 1 diabetes is typical of a Th1 pattern immune response, with the inflammatory cytokines IL-2, TNF-α, and IFN-γ being secreted in high quantities (178). The treatment of type I diabetes mellitus was conducted through the administration of hMSCs in a total of eight studies (74–80, 164). Among them, only one study (78) was conducted in humans and the other seven studies (74–77, 79, 80, 164) used mice as the experimental model. Among the studies conducted in mice, the fasting and post-prandial plasma glucose level, the C-peptide level, the rate of change in body weight, the serum insulin level, the total number of islets and the ratio of β and α cells per islet were the outcomes used by most animal studies selected in this systematic review to assess the potential of hMSCs administration for the treatment of type 1 diabetes mellitus. In the human clinical trial selected, the primary study endpoints used were: feasibility of the stem cell therapy; safety of the therapy through 24 months post-treatment; and the preliminary evaluation of the efficacy of the therapy for improving β cell function through 24 weeks. The secondary study endpoint used was the evidence of the efficacy of the therapy in modulating autoimmunity. We propose that the levels of glycated hemoglobin is the most appropriate primary endpoint for future clinical trials as this endpoint give us an overall picture of the average serum glucose levels over a period of weeks or months. In addition, we propose that secondary endpoints such as weight gain, occurrence of episodes of hypoglycemia, systolic and diastolic blood pressure and the level of circulating lipids should be used in conjunction with the primary endpoint selected to identify the existence of multiple effects associated with hMSC administration in the pathological course of type 1 diabetes mellitus. Regarding the source of hMSCs, in four studies hMSCs were isolated from the bone marrow (77, 79, 80, 164), in three (75, 76, 78) the umbilical cord was used as the source of hMSCs and in only one study (74) hMSCs were isolated from the adipose tissue. The administration of hMSCs affected both clinical and laboratory parameters of type I diabetes. In most of the studies selected, the administration of hMSCs resulted in a decrease in the blood glucose level (74–80) and in an increase in both the survival rates (75) and in the insulin level in the blood (75, 77, 79, 80). Furthermore, treatment with hMSCs delayed the onset of the disease (76, 164), reduced the weight loss resulting from the disease (74), inhibited insulitis in islets (75, 76, 164) and increased pancreatic islet number and function (74–78, 80).

Among the mechanisms proposed by the articles selected are included effects in the proliferation and differentiation of immune cells and changes in the expression pattern of growth factors, cytokines, enzymes, prostaglandins and surface receptors. A reduction in the levels of pro-inflammatory cytokines such as TNF-α (74, 75, 76), IFN-γ (75, 76, 77, 80), CCL2 (75), IL-1β (75), IL-2 (76, 77, 80), and IL-17 (75) and an increase in the expression of immunoregulatory cytokines such as IL-4 (74, 75), IL-10 (74, 75, 80), and IL-13 (74) was observed by the majority of the articles selected. In addition, in a study conducted by Wen et al. (80), the expression of the IL-2 receptor was also found to be decreased after the administration of hMSCs. In other studies, the expression of PGE2 (80) and growth factors such as TGF-β (75, 80), VEGF (80), and HGF (80) increased after hMSCs administration. Furthermore, according to Sun et al. (74), hMSCs exert an anti-apoptotic effect in pancreatic islets through the upregulation of the anti-apoptotic proteins XIAP, Bcl-xL, and Bcl-2 and the downregulation of the anti-apoptotic protein caspase 3. As a consequence, some studies reported a decrease in the amount of apoptotic cells when hMSCs were present (74, 80). Finally, a study conducted by Sun et al. (74) demonstrated that the expression of important transcription factors in islet development and differentiation such as Ngn3 and Pax6 was upregulated in pancreatic islets due to the presence of hMSCs. On the other hand, in a study by Zhang and Dou (79), hMSCs were differentiated into islet-like cells and their characteristics were compared to those of fetal pancreatic islets. This study demonstrated that the islet-like cells expressed the pancreatic islet cells-related genes pdx1, ngn3, pax4, neuroD1, nkx2.2, nkx6.1, PCSK1, insulin, glucagon, SST, and PP at levels similar to the expression profile of fetal pancreatic islets. Regarding the effects of hMSCs on the proliferation and differentiation of immune cells, some studies reported a stimulatory effect on the proliferation of CD4^+^CD25^+^Foxp3^+^ Treg (75, 76, 80, 164) cells while other results showed that the presence of hMSCs was found to be associated with the inhibition in the clonal expansion of CD4^+^IFN-γ^+^ Th1 (75) and CD4^+^IL17^+^ Th17 (75) cells. In addition, a study conducted by Tsai et al. (75) demonstrated that the administration of hMSCs inhibited the proliferation of CD11c^+^ dendritic cells in non-obese diabetic mice. Finally, a decrease in the infiltration of inflammatory T cells (77) and an increase in the proportion ofCD4^+^CD25^+^Foxp3^+^ Tregs (164) in the pancreatic islets were also observed. Table 2 summarizes the methodology employed and the results obtained in the studies selected in this systematic review regarding the effects of the administration of hMSCs for the treatment of type I diabetes mellitus. It is possible that the secretion of immunomodulatory cytokines and growth factors such as IL-4, IL-10, IL-13, TGF-β, PGE2, VEGF, and HGF by the hMSCs administered plays a crucial role in the inhibition in the development of the insulitis process mediated by CD4^+^ lymphocytes, as suggested by the inhibition in the clonal expansion of Th1 and Th17 lymphocytes after hMSCs administration. In addition, the stimulatory effect in the proliferation of Treg cells observed after hMSCs administration may exert an inhibitory effect in the proliferation and activation of dendritic cells, in clonal expansion of both T4^+^ and T8^+^ lymphocytes and in the infiltration of inflammatory T cells in pancreatic islets. Therefore, the decrease in the levels of pro-inflammatory cytokines such as TNF-α, IFN-γ, CCL2, IL-1β, IL-2, IL-17 observed by some of the studies selected can also be successfully explained by the inhibitory effects that hMSCs exert on inflammatory cells.

**Table 2:** List of *in vivo* studies in which the therapeutic potential of the administration of hMSCs for the treatment of type I diabetes mellitus was evaluated and the results obtained.

**References**	**Autoimmune disease**	**Source of hMSC**	**Variables**	**Experimental model**	**Clinical and laboratory effects**	**Mechanism proposed**
(164)	Type I diabetes mellitus	Bone marrow	None	Mice	↓Disease onset ↓Insulitis in the pancreas	↑Tregs in the pancreas
(74)	Type I diabetes mellitus	Adipose tissue	Administration of adipose tissue-derived MSCs overexpressing betatrophin Administration of adipose tissue-derived MSCs alone	Mice	↓Hyperglycemia ↓Weight loss Effectiveness of adipose tissue-derived MSCs overexpressing betatrophin > Effectiveness of adipose tissue-derived MSCs alone	↑Islet proliferation ↑Ngn3 transcription factor ↑Pax6 transcription factor ↑Islet production of insulin ↑β-cells ratio ↑IL-4 ↑IL-10 ↑IL-13 ↓TNF-α ↓NADP-cytochrome P450 reductase ↑XIAP in the islets ↑Bcl-xL in the islets ↑Bcl-2 in the islets ↓Caspase-3 in the islets ↓Apoptotic cells
(75)	Type I diabetes mellitus	Umbilical cord stroma	None	Mice	↑Survival rates ↓Blood glucose ↑Serum insulin levels ↑Glucose tolerance ↑C-peptide ↓Insulitis	↓Th1 cells ↓Th17 cells ↑Tregs ↓Dendritic cells ↓IFN-γ in the serum ↓IL-1β in the serum ↓TNF-α in the serum ↓CCL2 in the serum ↓IL-17 in the serum ↑IL-4 in the serum ↑IL-10 in the serum ↑TGF-β1 ↑Intact islets ↑Insulin-producing cells devided from the differentiation of umbilical cord stroma-derived MSCs
(76)	Type I diabetes mellitus	Umbilical cord stroma	Administration of umbilical cord-derived MSCs before the onset of type I diabetes	Mice	↓Disease onset ↑Fasting C-peptide ↓Insulitis	↑CD4^+^CD25^+^Foxp3^+^ Tregs ↓IL-2 ↓IFN-γ ↓TNF- α ↑Islet β-cells
			Treatment of type I diabetes with umbilical cord-derived MSCs after the onset of the disease		↓Fasting plasma glucose ↓Fed blood glucose ↑Fasting C-peptide ↓Insulitis	
(77)	Type I diabetes mellitus	Bone marrow	None	Mice	↓Hyperglycemia ↓Area under the glycemia curve ↓Fasting glycemia ↑Serum insulin	↑Islet β-cells function ↓CD3^+^ cells ↓Islet infiltration ↑Larger pancreatic islets ↓IL-2 in the pancreas ↓IFN-γ in the pancreas ↓IL-4 in the pancreas
(79)	Type I diabetes mellitus	Bone marrow	None	Mice	↓Hyperglycemia ↑Insulin production ↑Human insulin in mice ↑C-peptide production	↑Nestin ↑Pdx1 transcription factor ↑Ngn3 transcription factor ↑Pax4 transcription factor ↑NeuroD1 transcription factor ↑Nkx2.2 transcription factor ↑Nkx6.1 transcription factor ↑PCSK1 gene ↑Insulin gene ↑Glucagon gene ↑PCSK1 gene ↑PP gene
(80)	Type I diabetes mellitus	Bone marrow	None	Mice	↓Hyperglycemia ↑Insulin levels	↓Fas in human islets ↓MiR-375 in human islets ↓PBMC activation ↓PBMC proliferation ↓IL-2 ↓IFN-γ ↓IL-2 receptor ↑HGF ↑IL-10 ↑VEGF ↑PGE-2 ↑TGF-β ↑Treg function ↓Islet β-cells apoptosis ↑Islet β-cells function against inflammatory cytokines ↓Immune reaction against transplanted islets after humanization of mice
(78)	Type I diabetes mellitus	Umbilical cord stroma	None	Humans	↓Post-prandial plasma glucose ↓Hemoglobin HbA1c ↑Fasting C peptide ↑C-peptide/glucose ratio	↑Islet β-cells function

*Both the methodology employed and the results obtained by each article are represented in this table. Ngn3, neurogenin-3; Pax4, paired box gene 4; Pax6, paired box gene 6; IL-13, interleukin 13; XIAP, X-linked inhibitor of apoptosis protein; Bcl-xL, B-cell lymphoma-extra large; Bcl-2, B-cell lymphoma 2; Pdx1, pancreatic and duodenal homeobox 1; NeuroD1, neurogenic differentiation 1; Nkx2.2, homeobox protein Nkx-2.2; Nkx6.1, homeobox protein Nkx-6.1; PCSK1, proprotein convertase 1; PP, protein phosphatase; Fas, first apoptosis signal receptor; PBMC, peripheral blood mononuclear cell*.

#### Treatment of type i diabetes mellitus

Type 1 diabetes mellitus is a chronic metabolic disease characterized by an insulin deficiency caused by the cellular-mediated autoimmune destruction of the β-cells of the pancreas (175). The process of destruction of pancreatic β cells, called insulitis, is a consequence of an immunological attack mediated by lymphocytes, macrophages and NK cells and leads to a permanent hyperglycemia and the need for exogenous insulin replacement (176). CD8^+^ T lymphocytes are the predominant type of immune cell responsible for the insulitis process, but the presence of CD4^+^ T lymphocytes and B lymphocytes can also be detected in the lymphocytic infiltrate in pancreatic islets (177). Furthermore, the cytokine secretion profile during the development of type 1 diabetes is typical of a Th1 pattern immune response, with the inflammatory cytokines IL-2, TNF-α, and IFN-γ being secreted in high quantities (178). The treatment of type I diabetes mellitus was conducted through the administration of hMSCs in a total of eight studies (74, 75, 76, 77, 78, 79, 80, 164). Among them, only one study (78) was conducted in humans and the other seven studies (74, 75, 76, 77, 79, 80, 164) used mice as the experimental model. Among the studies conducted in mice, the fasting and post-prandial plasma glucose level, the C-peptide level, the rate of change in body weight, the serum insulin level, the total number of islets and the ratio of β and α cells per islet were the outcomes used by most animal studies selected in this systematic review to assess the potential of hMSCs administration for the treatment of type 1 diabetes mellitus. In the human clinical trial selected, the primary study endpoints used were: feasibility of the stem cell therapy; safety of the therapy through 24 months post-treatment; and the preliminary evaluation of the efficacy of the therapy for improving β cell function through 24 weeks. The secondary study endpoint used was the evidence of the efficacy of the therapy in modulating autoimmunity. We propose that the levels of glycated hemoglobin is the most appropriate primary endpoint for future clinical trials as this endpoint give us an overall picture of the average serum glucose levels over a period of weeks or months. In addition, we propose that secondary endpoints such as weight gain, occurrence of episodes of hypoglycemia, systolic and diastolic blood pressure and the level of circulating lipids should be used in conjunction with the primary endpoint selected to identify the existence of multiple effects associated with hMSC administration in the pathological course of type 1 diabetes mellitus. Regarding the source of hMSCs, in four studies hMSCs were isolated from the bone marrow (77, 79, 80, 164), in three (75, 76, 78) the umbilical cord was used as the source of hMSCs and in only one study (74) hMSCs were isolated from the adipose tissue. The administration of hMSCs affected both clinical and laboratory parameters of type I diabetes. In most of the studies selected, the administration of hMSCs resulted in a decrease in the blood glucose level (74–80) and in an increase in both the survival rates (75) and in the insulin level in the blood (75, 77, 79, 80). Furthermore, treatment with hMSCs delayed the onset of the disease (76, 164), reduced the weight loss resulting from the disease (74), inhibited insulitis in islets (75, 76, 164) and increased pancreatic islet number and function (74–78, 80).

Among the mechanisms proposed by the articles selected are included effects in the proliferation and differentiation of immune cells and changes in the expression pattern of growth factors, cytokines, enzymes, prostaglandins and surface receptors. A reduction in the levels of pro-inflammatory cytokines such as TNF-α (74, 75, 76), IFN-γ (75, 76, 77, 80), CCL2 (75), IL-1β (75), IL-2 (76, 77, 80), and IL-17 (75) and an increase in the expression of immunoregulatory cytokines such as IL-4 (74, 75), IL-10 (74, 75, 80), and IL-13 (74) was observed by the majority of the articles selected. In addition, in a study conducted by Wen et al. (80), the expression of the IL-2 receptor was also found to be decreased after the administration of hMSCs. In other studies, the expression of PGE2 (80) and growth factors such as TGF-β (75, 80), VEGF (80), and HGF (80) increased after hMSCs administration. Furthermore, according to Sun et al. (74), hMSCs exert an anti-apoptotic effect in pancreatic islets through the upregulation of the anti-apoptotic proteins XIAP, Bcl-xL, and Bcl-2 and the downregulation of the anti-apoptotic protein caspase 3. As a consequence, some studies reported a decrease in the amount of apoptotic cells when hMSCs were present (74, 80). Finally, a study conducted by Sun et al. (74) demonstrated that the expression of important transcription factors in islet development and differentiation such as Ngn3 and Pax6 was upregulated in pancreatic islets due to the presence of hMSCs. On the other hand, in a study by Zhang and Dou (79), hMSCs were differentiated into islet-like cells and their characteristics were compared to those of fetal pancreatic islets. This study demonstrated that the islet-like cells expressed the pancreatic islet cells-related genes pdx1, ngn3, pax4, neuroD1, nkx2.2, nkx6.1, PCSK1, insulin, glucagon, SST, and PP at levels similar to the expression profile of fetal pancreatic islets. Regarding the effects of hMSCs on the proliferation and differentiation of immune cells, some studies reported a stimulatory effect on the proliferation of CD4^+^CD25^+^Foxp3^+^ Treg (75, 76, 80, 164) cells while other results showed that the presence of hMSCs was found to be associated with the inhibition in the clonal expansion of CD4^+^IFN-γ^+^ Th1 (75) and CD4^+^IL17^+^ Th17 (75) cells. In addition, a study conducted by Tsai et al. (75) demonstrated that the administration of hMSCs inhibited the proliferation of CD11c^+^ dendritic cells in non-obese diabetic mice. Finally, a decrease in the infiltration of inflammatory T cells (77) and an increase in the proportion ofCD4^+^CD25^+^Foxp3^+^ Tregs (164) in the pancreatic islets were also observed. Table 2 summarizes the methodology employed and the results obtained in the studies selected in this systematic review regarding the effects of the administration of hMSCs for the treatment of type I diabetes mellitus. It is possible that the secretion of immunomodulatory cytokines and growth factors such as IL-4, IL-10, IL-13, TGF-β, PGE2, VEGF, and HGF by the hMSCs administered plays a crucial role in the inhibition in the development of the insulitis process mediated by CD4^+^ lymphocytes, as suggested by the inhibition in the clonal expansion of Th1 and Th17 lymphocytes after hMSCs administration. In addition, the stimulatory effect in the proliferation of Treg cells observed after hMSCs administration may exert an inhibitory effect in the proliferation and activation of dendritic cells, in clonal expansion of both T4^+^ and T8^+^ lymphocytes and in the infiltration of inflammatory T cells in pancreatic islets. Therefore, the decrease in the levels of pro-inflammatory cytokines such as TNF-α, IFN-γ, CCL2, IL-1β, IL-2, IL-17 observed by some of the studies selected can also be successfully explained by the inhibitory effects that hMSCs exert on inflammatory cells.

#### Treatment of systemic lupus erythematosus and systemic sclerosis

Systemic lupus erythematosus is a chronic, multisystemic autoimmune disease characterized by the production of autoantibodies, formation and deposition of immunocomplexes, inflammation in various organs and tissue damage (179). The disease progresses with polymorphic clinical manifestations and periods of exacerbation and remission (180). In this disease, the imbalance that occurs in the regulation of the immune response results in the production of several auto-reactive antibodies, which react against the components of the nucleus such as the DNA, ribonucleoproteins and histones, giving rise to immunecomplexes (181). The antigens released by this process of apoptosis increases the production of autoreactive antibodies. The mechanism of production of autoreactive antibodies observed in patients with systemic lupus erythematosus occurs through the recognition of apoptotic fragments by B cells through the B cell receptor (BCR); the BCR recognizes the fragments resulting from the apoptotic process and internalizes them into the B lymphocyte (182). The fragment is then processed and associated with a MHC class II molecule. This complex is subsequently presented by B lymphocytes to CD4^+^ T lymphocytes, which recognizes the antigen previously presented and initiates the production of cytokines and induces the differentiation of these B lymphocytes into plasma cells. These plasmocytes are responsible for secreting specific autoreactive antibodies against the components of the cell nucleus (183). Finally, the binding of the antibody to the antigen culminates in the formation of immunocomplexes. In eight studies (81–84, 159–162), hMSCs were administered for the treatment of systemic lupus erythematosus, all of them used mice as the experimental model. Surprisingly, the bone marrow was chosen as the source of hMSCs in only two (161, 162) out of these eight studies analyzed. hMSCs were also isolated from the adipose tissue (83, 159) and from the dental pulp (84, 162) in two studies each. The umbilical cord was used as the source of hMSCs in only one study (82) and in two studies (81, 160) hMSCs were obtained from the differentiation of embryonic stem cells.

The cumulative survival rate, the cumulative incidence of proteinuria, the urinary level of albumin, and the serum levels of creatinine, albumin, blood urea nitrogen and anti-double-stranded DNA antibodies were the outcomes used by most studies selected in this systematic review to assess the potential of hMSCs administration for the treatment of systemic lupus erythematosus. Because there were no human clinical trials among the studies selected, it was not possible to identify primary endpoints commonly used to evaluate the effectiveness of hMSCs administration for the treatment of systemic lupus erythematosus in humans. Systemic lupus erythematosus is very heterogeneous disease, being able to affect virtually every organ system and culminating in the development of a wide variety of clinical and biologic manifestations (180). As a result, choosing a single endpoint in systemic lupus erythematosus clinical trials is not an easy task, as it is very difficult to capture the overall systemic lupus erythematosus disease activity across multiple systems. We, therefore, recommend the use of systemic lupus erythematosus activity scores such as the Systemic Lupus Erythematosus Disease Activity Index (SLEDAI) (184), the European Consensus Lupus Activity Measurement (ECLAM) (185), and British Isles Lupus Assessment Group index (BILAG) (186). These scores are composed of a combination of several variables and are able to capture the overall systemic lupus erythematosus disease activity across all possible organ system manifestations. In addition, exploratory endpoints such as the serum levels of cytokines and autoantibodies can be used in combination with the primary endpoint in order to assess the immunomodulatory activity of hMSCs administration for the treatment of systemic lupus erythematosus. In the majority of the studies selected, the treatment of systemic lupus erythematosus resulted in a reduction in the severity (159, 160) of the disease and in an increase the survival rates observed (81, 82, 84, 159, 161). In addition, other studies reported that the administration of hMSCs reduced interstitial inflammation (160) and attenuated glomerulonephritis (161) and other kidney injuries (82–84), as evidenced by the decrease in proteinuria (82, 159–161), blood urea nitrogen (159), serum creatine (160), and glomerular IgG deposition (159).

Regarding the mechanisms proposed for the action of hMSCs, most studies demonstrated that the administration of hMSCs increased the levels of immunoregulatory cytokines such as IL-10 (82, 159) and IL-4 (82, 159) and reduced the levels of pro-inflammatory ckytokines such as IFN-γ (82), TNF-α (82, 160), IL-2 (82), IL-6 (82, 160), IL-12 (81, 82), and IL-17 (84, 162). A decrease in the proliferation of T lymphocytes (81, 82) and splenocytes (82) following the use of hMSCs was also observed. In particular, some studies reported that the treatment with hMSCs resulted in the inhibition in the clonal expansion of CD4^+^IL-17^+^ Th17 (83, 84), CD4^+^IFN-γ^+^ Th1 (83), and CD4^+^ICOS^+^CD44^+^ Tfh (161) cells and in the stimulation in the proliferation of CD4^+^CD25^+^FoxP3^+^ Treg (81, 83, 160) cells. Furthermore, effects in the proliferation and differentiation of B cells were also observed. For instance, Park et al. (83) reported the occurrence of a stimulatory effect on the expansion CD1d^hi^CD5^+^and CD1d^hi^CD5^+^IL-10^+^Breg cells mediated by hMSCs. This study also described that the administration of hMSCs inhibited the proliferation of both B220^+^CD23^high^CD21^low^ FOB cells and B220^−^CD138^+^IgD^−^ plasma cells and stimulated the expansion of B220^+^CD23^low^CD21^high^ MZB cells. Park et al. (83) also demonstrated that mice treated with human hMSCs showed significantly decrease in the size and number of germinal centers. Additionally, a study conducted by Jang et al. (161) demonstrated that the administration of hMSCs decreased the proportions of B220^+^GL7^+^GC B cells and B220^lo^CD138^+^ plasma cells, and inhibited the infiltration of these plasma cells into the kidneys. As a consequence of the suppression in both the development of Tfh cells and the subsequent activation of humoral immune components, a decrease in the levels of the autoantibodies to components of the cell nucleus that are usually associated with the development of systemic lupus erythematosus was observed by the majority of the studies selected. Finally, a study by Kimbrel et al. (81) demonstrated that hMSCs suppressed the expression of CD83 in dendritic cells and their secretion of IL-12, both of which are involved in the maturation and activation process of this cell type and are crucial to their ability to properly deliver signals to T cells.

Systemic sclerosis is also an autoimmune systemic disease, characterized by inflammation and vascular hyperreactivity of the microcirculation and macrocirculation associated with excessive deposition of collagen in the tissues, resulting in fibrosis in the skin and in internal organs (187). Clinically, the disease is characterized by inflammatory, fibrotic and atrophic alterations, along with proliferative endarteritis and obstructive capillary lesions compromising the skin, musculoskeletal system and internal organs, particularly the heart, kidneys, lungs and gastrointestinal tract (188). The main cause of death from systemic sclerosis is related to its pulmonary involvement, which often results in pulmonary hypertension. The exacerbate production of the cytokines IL-4 and IL-13 is a result of the activation of T cells by antigens and the subsequent induction of a Th2 response, which stimulates the process of fibrosis (189). Autoantibodies are also produced in high quantities due to the activation of B cells, which adopts a profibrotic phenotype. Finally, macrophages in perivascular infiltrates are also activated, leading to the production of CCL2, TGF-β, and platelet-derived growth factor (PDGF), all of which promote fibrosis and fibroproliferation (190). Only one (85) of the studies analyzed applied hMSCs for the treatment of systemic sclerosis. This study was conducted in humans and used the umbilical cord as the source of hMSCs. In this study, the primary study endpoints used were: the modified Rodnan skin score; and variables associated with interstitial lung disease such as the diffusing capacity of the lung for carbon monoxide and the forced vital capacity. The serum levels of TGF-β, VEGF and anti-SCL70 IgG antibody were used as additional study endpoints as they provide a strong evidence of the efficacy of the therapy in modulating autoimmunity and decreasing the levels of profibrotic mediators. The modified Rodnan skin score is the primary endpoint used almost universally in systemic sclerosis clinical trials. However, the Rodnan skin score does not describe the progression of the disease across multiple organ systems and is vulnerable to observation bias in single-arm open label trials as this method is based on interpretation by both physicians and patients. Therefore, we propose that the disease-free survival rate should be considered the most appropriate primary endpoint in clinical trials to assess the effectiveness of hMSCs administration for the treatment of systemic sclerosis as this endpoint is able to identify the occurrence of the disease in multiple organs and is less vulnerable to bias. Furthermore, exploratory endpoints such as the serum level of TGF-β and PDGF should used in conjunction with the primary endpoint selected in order to allow the researchers to assess the efficacy of the hMSCs administration in modulating autoimmunity. The study selected demonstrated that the administration of hMSCs resulted in an improvement in both the modified Rodnan Skin Score and lung function. Furthermore, a decrease in the serum levels of inflammatory markers and profibrotic mediators such as TGF-β and VEGF and in level of the anti-Scl70 autoantibody was also observed during follow up. Table 3 summarizes the methodology employed and the results obtained in the studies selected in this systematic review regarding the effects of the administration of hMSCs for the treatment of systemic lupus erythematosus and systemic sclerosis.

**Table 3:** List of *in vivo* studies in which the therapeutic potential of the administration of hMSCs for the treatment of systemic lupus erythematosus and systemic sclerosis was evaluated and the results obtained.

**References**	**Autoimmune disease**	**Source of hMSC**	**Variables**	**Experimental model**	**Clinical and labortory effects**	**Proposed mechanisms for the *in vivo* action of MSCs**
(159)	Systemic lupus erythematosus	Adipose tissue	Administration of adipose tissue-derived MSCs alone Administration of adipose tissue-derived MSCs overexpressing CTLA4Ig	Mice	↑Survival rates ↓Clinical score ↓Proteinuria ↓Blood urea nitrogen Effectiveness MSCs overexpressing CTLA4Ig = effectiveness MSCs alone	↓Glomerular IgG deposition ↑IL-10 ↑IL-4
(81)	Systemic lupus erythematosus	Embryonic stem cells	None	Mice	↑Survival rates	↓T-cell proliferation ↓CD83^+^ dendritic cells ↓IL-12 ↑CD4^+^CD25^+^FoxP3^+^ Tregs
(160)	Systemic lupus erythematosus	Embryonic stem cells	None	Mice	↓Clinical score ↓Interstitial inflammation ↓Protein cast deposition ↓Proteinuria ↓Serum creatine	↓TNF-α ↓IL-6 ↑CD4^+^/CD25^+^ Tregs ↓Infiltration of CD3^+^ lymphocytes in the kidneys
(161)	Systemic lupus erythematosus	Bone marrow	None	Mice	↑Survival rates ↓Glomerulonephritis ↓Proteinuria	↓Autoantibodies ↓CD4^+^CXCR5^+^PD-1^+^ follicular helper T cells ↓Infiltration of B220^lo^CD138^+^ plasma cells into the kidney ↓Differentiation of naive CD4^+^ T cells toward Tfh cells
(82)	Systemic lupus erythematosus	Umbilical cord blood	None	Mice	↓Renal injury ↑Survival rates ↓Proteinuria	↓Anti-dsDNA autoantibody ↓IFN-γ ↓IL-2 ↓TNF-α ↓IL-6 ↓IL-12 ↑IL-4 ↑IL-10 ↓T lymphocytes proliferation ↓Splenocytes proliferation
(83)	Systemic lupus erythematosus	Adipose tissue	None	Mice	↓Renal injury	↓Serum anti-double-stranded autoantibody ↓CD4^+^ICOS^+^CD44^+^ follicular helper T cells in spleen ↓CD4^+^IFN-γ^+^ Th1 cells in spleen ↓CD4^+^ IL-17^+^ Th17 cells in spleen ↑CD1d^hi^CD5^+^ Bregs in spleen ↑CD1d^hi^CD5^+^IL-10^+^ Bregs in spleen ↑CD4^+^Foxp3^+^Tregs cells ↓B220^+^CD23^high^CD21^low^ FOB cells ↓B220^−^CD138^+^IgD^−^ plasma cells ↑B220^+^CD23^low^CD21^high^ MZB cells ↓Size and number of germinal centers
(84)	Systemic lupus erythematosus	Dental pulp	None	Mice	↑Survival rates ↓Renal injury	↓Viability of T-cells ↓CD4^+^IL-17^+^ Th17 cells ↓Serum autoantibodies ↓IL-17 ↓T-cells
(162)	Systemic lupus erythematosus	Bone marrow Dental pulp	None	Mice	↑Bone density and structure	↑Osteoclastogenesis ↓Osteoblastogenesis ↓IL-17 in the recipient bone marrow
(85)	Systemic sclerosis	Umbilical cord stroma	None	Humans	↓Modified Rodnan skin score ↑Diffusing capacity of the lung for carbon monoxide ↑Forced vital capacity	↓Serum anti-Scl70 autoantibody ↓Serum TGF-β ↓Serum VEGF

*Both the methodology employed and the results obtained by each article are represented in this table. Scl70, anti-topoisomerase I after the type I topoisomerase target; MZB cells, marginal zone B-cells; FOB cells, follicular B cells; dsDNA, double stranded DNA*.

#### Treatment of autoimmune disorders of the joints

Rheumatoid arthritis is an autoimmune, inflammatory, systemic and chronic disease characterized by peripheral synovitis and several extra-articular manifestations. The typical clinical manifestations of rheumatoid arthritis include pain and swelling of the joints (191). Regarding the inflammatory process that typically occurs in rheumatoid arthritis, blood cells and inflammatory mediators migrate into the joints, resulting in synovial hyperplasia. As a result of this process, both the synovial membrane of the diarthrodial joints and other joint structures, cartilage and bone are damaged (191). In addition to the invasion of the entire joint, these pro-inflammatory cells also invade other tissues, such as ligaments, tendons and bone, causing similar lesions (191). The invasion of the cartilage by pro-inflammatory cells leads to degradation of type II collagen by matrix metalloproteinases, and by other enzymes produced by synovial cells and chondrocytes when stimulated by inflammatory cytokines such as TNF-α, IL-1, IL-6, and IL-17, secreted by cells from the inflammatory infiltrate (192). hMSCs were used for the treatment of rheumatoid arthritis in 17 studies (72, 86–97, 152, 153, 157, 158). Among them, 14 (72, 86, 87, 89–94, 96, 152, 153, 157, 158) used mice as the experimental model, three (88, 95, 97) used rats and no study was conducted in humans. Regarding the source of the hMSCs used, the adipose tissue was chosen as the source of hMSCs by the majority of studies analyzed. In a total of seven studies (86, 87, 94, 96, 152, 153, 158) hMSCs were isolated from the adipose tissue while the umbilical cord was used as the source of hMSCs in six studies (89, 91, 93, 95, 97, 158) and in only four studies hMSCs were obtained from the bone marrow (72, 92, 95, 158). Furthermore, hMSC were isolated from the placenta (88), gingival (90) and menstrual blood (72) in one study each. Finally, in one study (157), hMSCs were obtained from the directed differentiation of embryonic stem cells.

The arthritis severity score, the incidence of arthritis, the bone erosion score, the synovial hyperplasia score, the cell infiltration score, the cartilage degradation score and the serum levels of anti-mouse type II collagen antibody, C-telopeptide I, and C-telopeptide II were the outcomes used by most studies selected in this systematic review to assess the potential of hMSCs administration for the treatment of rheumatoid arthritis. Due to the fact that there were no human clinical trials among the studies selected, it was not possible to identify primary endpoints commonly used to evaluate the effectiveness of hMSCs administration for the treatment of rheumatoid arthritis in humans. In rheumatoid arthritis clinical trials, endpoints commonly used to assess the efficacy of a treatment are the American College of Rheumatology 20% improvement criteria (ACR20), ACR50, and ACR70 response rates (193), and the 28-joint disease activity score (DAS28) (194). All of these endpoints are effective when used in large clinical trials. When a clinical trial is composed of a small group of patients, however, we recommend the use of endpoints that are composed of continuous variables such as the DAS28 and hybrid ACR response as they are more sensitive to change than the ACR20, ACR50, and ACR70 response criteria. In addition, it is desirable to include exploratory endpoints such as the serum levels of anti and pro-inflammatory cytokines and the proportion of inflammatory cells in order to evaluate the influence of the hMSCs administration in the inflammatory process of the disease.

A reduction in both the severity of the disease (72, 86–98, 152, 153, 157, 158) and in the histopathology scores (88, 90, 93, 96, 158) after treatment with hMSCs was observed by the majority of studies. Furthermore, a reduction in the incidence of the disease was also reported (152). As a result, the serum level of c-telopeptide of type II collagen, a marker of cartilage degradation, was found to be decreased following hMSCs administration (86). Administration of hMSCs had an inhibitory effect in the production of pro-inflammatory cytokines such as TNF-α (72, 88, 89, 96, 97, 152, 158), IFN-γ (88, 90, 152, 158), IL-1β (89, 96, 97, 158), IL-2 (152), IL-17 (90, 152), CCL5 (152), and CXCL2 (152) and a stimulatory effect in the secretion of anti-inflammatory cytokines such as IL-5 (72), IL-10 (72, 89, 94, 96, 152, 158), and IL-13 (72). In particular, the level of IL-6, a cytokine with both pro and anti-inflammatory properties, was found to be decreased following the use of hMSCs in some studies (91, 96, 158), while in another study a higher level of IL-6 was detected after treatment with hMSCs (72). Administration of hMSCs had also a stimulatory effect in the expression of TGF-β (94, 152, 158), IDO (72, 94, 157), PGE2 (72), PDL-1 (72), and activin A (72), as demonstrated by some studies. Furthermore, in a study conducted by Gu et al. (97), a decrease in the serum levels of the inflammatory marker VEGF and the procoagulant tissue factor (TF) and an increase in the level of the anticoagulant protein antithrombin was also observed. In addition, a study conducted by Shu et al. (88) demonstrated the administration of hMSCs exerted anti-oxidative effects by significantly increasing the levels of SOD, GSH-Px, T-AOC and reducing the level of MDA. Finally, administration of hMSCs also proved to be effective in reducing the levels of autoreactive antibodies against type II collagen (86, 96, 152).

**Figure 6:**
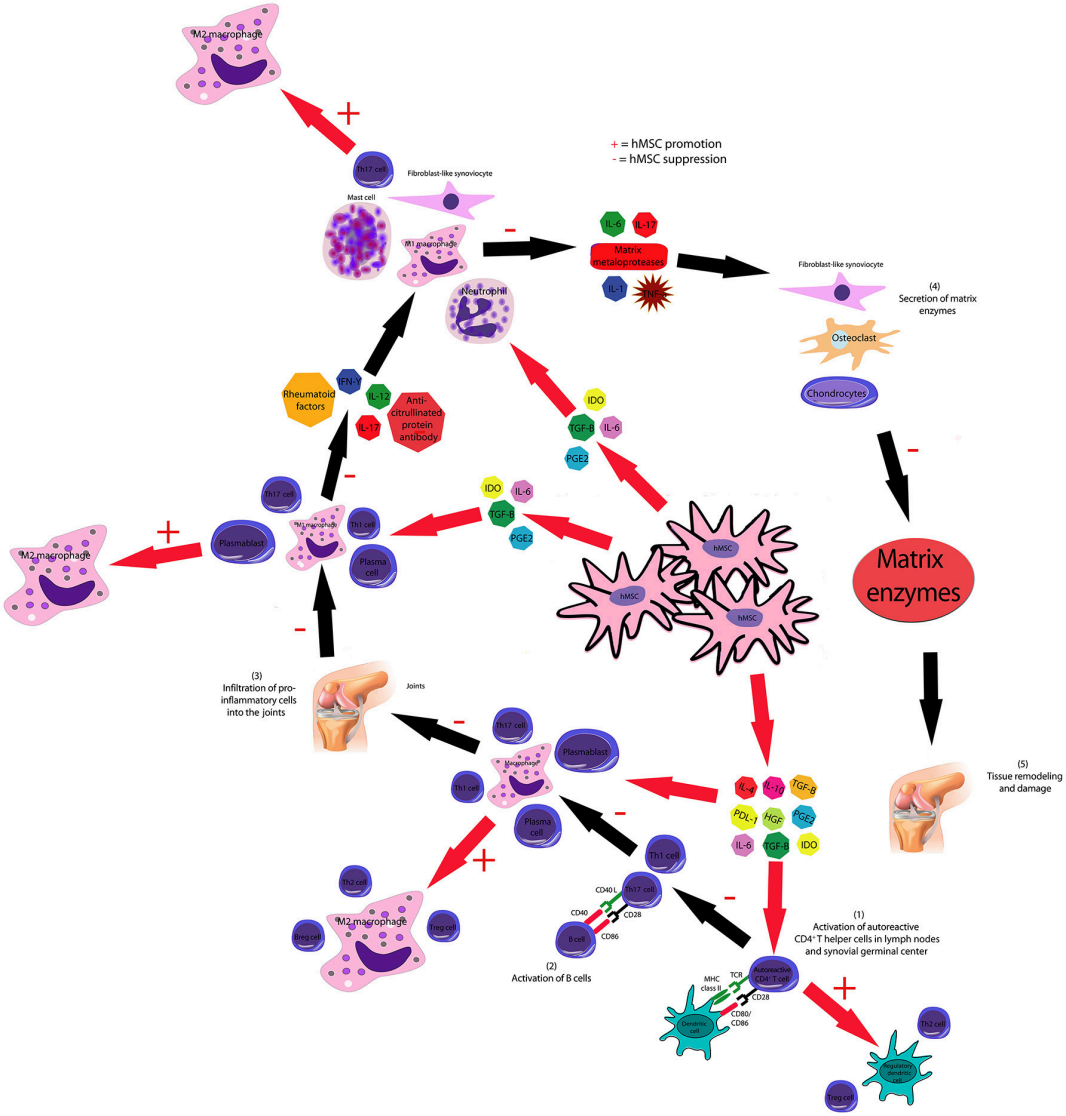
hMSCs inhibit the pathological course of rheumatoid arthritis through several mechanisms. hMSC-produced IL-4, IL-10, HGF, PGE2, PDL-1, and TGF-β inhibit the proliferation and activation of T and B cells and stimulate the generation of Breg, Treg, and Th2 lymphocytes. hMSCs inhibit the activation of dendritic cells and stimulate the generation of regulatory dendritic cells. hMSC-produced IL-6, IDO, PGE2, and TGF-β suppresses neutrophil respiratory burst, NK cell activation and macrophage polarization to M1, though favors M2 polarization. As a consequence, the secretion of matrix enzymes by chondrocytes, osteoclasts and fibroblast-like synoviocytes is also decreased.

Treatment with hMSCs had also significant effects in the proliferation and differentiation of immune cells. Findings commonly reported by the articles selected included an inhibition in the clonal expansion of Th17 (72, 91–94, 153), Th1 (72, 93, 94, 152, 157), and Tfh (93) cells and a stimulation in the proliferation of T cells with a regulatory phenotype, such as Treg cells (72, 86, 90, 93–97, 152, 157, 158) (CD4^+^CD25^+^Foxp3^+^) and Tr1 cells (87) (CD4^+^IL-4^+^IL10^+^). This effect in the clonal expansion of CD4^+^ T cells can be effectively explained by the dowregulation in the T-bet and GATA-3 genes following the administration of hMSCs, as observed in a study conducted by Choi et al. (86). In a study conducted by Lopez-Santalla et al. (153), however, increased numbers of Th17 cells coexpressing IL-10 were found in the draining lymph nodes of mice with established collagen-induced arthritis treated with hMSCs. This study also reported a decrease in the number of pathogenic CD4^+^GM-CSF^+^ T cells in the spleen and peripheral blood of mice with collagen-induced arthritis treated with hMSCs. An inhibition in the proliferation of CD8^+^IFN-γ^+^ T lymphocytes was also observed after the treatment with hMSCs. Finally, a study conducted by Shin et al. (89) demonstrated that the administration of hMSCs shifted the macrophage functional phenotype from the CD14^+^CD86^+^ M1 phenotype to the CD14^+^CD206^+^ M2 phenotype. In this study, lower levels of IL-1β and caspase-1 were also detected in supernatants of macrophages co-cultured with hMSCs, leading to a suppression in the activation of the NLRP3 inflammasome. It is possible to hypothesize that the reduction in the severity of the disease and in the histopathology scores observed after the treatment with hMSCs is a consequence of the ability of these cells to both inhibit the proliferation and activation of immune cells such as Tfh, Th1, Th17, and CD8 lymphocytes and M1 macrophages. As a result, the process of invasion of the cartilage by these pro-inflammatory cells is also inhibited, reducing the levels of inflammatory cytokines such as TNF-α, IFN-γ, IL-1β, IL-2, and IL-17 in this tissue and decreasing the degradation of type II collagen by matrix metalloproteinases produced by synovial cells and chondrocytes. In addition, hMSCs administration stimulated the proliferation of cells with regulatory phenotypes such as Treg and Tr1 lymphocytes and suppressed macrophage polarization to M1, though favors M2 polarization, increasing the levels of anti-inflammatory cytokines such as IL-5, IL-10, and IL-13 and reducing the inflammation necessary for the occurrence of the pathological process. The mechanisms proposed by this systematic review concerning the inhibition in the progression of the pathological process of rheumatoid arthritis mediated by hMSCs are represented in Figure 6.

Ankylosing spondylitis is an inflammatory disease that affects the connective tissues, characterized by inflammation of the joints, such as the hip, shoulders and other regions. Clinically, this inflammatory process is characterized by edema, pain and hyperthermia of joints (195). In the early phase, there is release of inflammatory cytokines, such as IL-1, IL-6, and TNF-α, which causes recruitment of inflammatory cells, especially macrophages (196). In a later and chronic phase, a deviation of the immunological pattern occurs, from a Th1 inflammatory response to a Th2 cellular response. In this case, previously secreted inflammatory cytokines are decreased and the secretion of IL-4, IL-10, and TGF-β increases, which inhibit the recruitment of macrophages and stimulates the proliferation of lymphocytes (197). At this stage, the autoantigen derived from the enthesial fibrocartilage is produced, and its presence induces the formation of syndesmophytes in the joint, culminating in ankylosis of the vertebral column. The treatment of ankylosing spondylitis was conducted with hMSCs in only one study (98) out of the 132 studies (33–164) selected. This study was carried out in humans and used the bone marrow as the source of hMSCs. In this study, the percentage of assessment in ankylosing spondylitis response criteria (ASAS)20 responders at the fourth week was chosen as the primary endpoint and the mean ASAS20 response duration was chosen as the secondary endpoint in order to assess both the induction of response and the maintenance of response following hMSCs administration. Indeed, both the proportion of patients who achieved ASAS20 and the mean ASAS20 duration are appropriate endpoints that can be used to evaluate the effectiveness of hMSCs administration in the treatment of ankylosing spondylitis. However, other endpoints such as the ASAS40 improvement criteria and the ASAS partial remission criteria should be used in conjunction with the ASAS20 improvement criteria as this endpoint can both exclude patients that achieved better results and underestimate the duration of effectiveness. In this study, a reduction in the parameters of the disease was observed after administration of hMSCs. Table 4 summarizes the methodology employed and the results obtained in the studies selected in this systematic review regarding the effects of the administration of hMSCs for the treatment of autoimmune disorders of the joints.

**Table 4:** List of *in vivo* studies in which the therapeutic potential of the administration of hMSCs for the treatment of autoimmune disorders of the joints was evaluated and the results obtained.

**References**	**Autoimmune disease**	**Source of hMSC**	**Variables**	**Experimental model**	**Clinical and laboratory effects**	**Proposed mechanisms for the *in vivo* action of MSCs**
(80)	Rheumatoid arthritis	Bone marrow Menstrual blood	None	Mice	↓Clinical score Effectiveness MSCs from bone marrow > effectiveness MSCs from menstrual blood	↓CD8^+^IFN-γ+ cells ↓CD4^+^IFN-γ+ Th1 cells ↓Th17 cells in lymph nodes ↑CD4^+^IL4^+^IL10^+^ Treg cells ↑Tregs ↑IDO ↑PD-L1 ↑PGE2 ↑Activin A ↑IL-5 ↑IL-10 ↑IL-13 ↑IL-6 ↓TNF-α (bone marrow MSCs) ↑TNF-α (menstrual blood MSCs) ↓Proinflammatory T cell frequency in the lymph nodes
(152)	Rheumatoid arthritis	Adipose tissue	None	Mice	↓Clinical score ↓Disease incidence	↓CD4^+^TNF-α^+^IFN-γ^+^ Th1 cells ↑CD4^+^IL-10^+^ Treg cells ↑IL-10 in lymph nodes and joints ↑CD4^+^CD25^+^FoxP3^+^ Treg cells ↓CCL5 expression ↓TNF-α ↓IFN-γ ↓IL-2 ↓IL-17 ↑IL-10 ↑TGF-β1 ↓Type II collagen autoantibody ↓CXCL2
(153)	Rheumatoid arthritis	Adipose tissue	None	Mice	↓Clinical score	↓Pathogenic GM-CSF^+^CD4^+^ T cells in the spleen and peripheral blood ↑FoxP3^+^CD4^+^ T cells in the draining lymph nodes ↑IL10^+^IL17^−^CD4^+^ T cells in the draining lymph nodes ↑IL-10^+^ Th17 cells in the draining lymph nodes
(157)	Rheumatoid arthritis	Embryonic stem cells	None	Mice	↓Clinical score	↑ CD4^+^FoxP3^+^ Treg cells ↑ CD4^+^IFN-γ^+^ Th1 cells ↑IDO1 gene
(158)	Rheumatoid arthritis	Bone marrow Adipose tissue Umbilical cord blood	None	Mice	↓Clinical score ↓Histopathology score Effectiveness bone marrow-derived MSCs = effectiveness adipose tissue-derived MSCs = effectiveness umbilical cord-derived MSCs	↓IL-1β ↓TNF-α ↓IL-6 ↓IFN-γ ↑IL-10 ↑TGF-β ↑Treg cells
(86)	Rheumatoid arthritis	Adipose tissue	Administration of adipose tissue-derived MSCs aloneAdministration of adipose tissue-derived MSCs overexpressing CTLA4Ig	Mice	↓Clinical score ↓Serum C-Telopeptide I Effectiveness MSCs overexpressing CTLA4Ig > effectiveness MSCs alone	↓Type II collagen autoantibody ↓T-bet expression in splenocytes ↓GATA-3 expression in splenocytes (*in vitro*) ↑Treg/Th17(CD4^+^CD25^+^FoxP3^+^/ CD4^+^CD25^+^RORγt) cells ratio
(87)	Rheumatoid arthritis	Adipose tissue	None	Mice	↓Clinical score ↓Disease progression ↓Bone destruction	↑CD25+Foxp3+CD4+ cells regulatory T cells in spleen and draining lymph nodes ↑CD4^+^IL-4^+^IL10^+^ Tr1 cells in spleen and draining lymph nodes
(89)	Rheumatoid arthritis	Umbilical cord bood	None	Mice	↓Clinical score	↑Shift from CD14^+^CD86^+^ M1 to CD14^+^CD206^+^ M2 macrophages ↓NLRP3 inflammasome-mediated IL-1β secretion ↓TNF-α ↓IL-1β ↓Caspase-1 ↑IL-10
(90)	Rheumatoid arthritis	Gingiva	None	Mice	↓Clinical score ↓Histopathology scores	↓IFN-γ ↓IL-17 ↑CD4^+^CD39^+^FoxP3^+^ Treg cells
(91)	Rheumatoid arthritis	Umbilical cord stroma	Administration of CD146^+^ MSCs Administration of CD146^−^ MSCs	Mice	↓Clinical score Effectiveness CD146^+^ MSCs > effectiveness CD146^−^ MSCs	↓IL-6 ↑Th17 cells
(92)	Rheumatoid arthritis	Bone marrow	Administration of MSCs transfected with recombinant minicircles encoding etanerceptAdministration of MSCs alone	Mice	↓Clinical score Effectiveness of MSCs transfected with recombinant minicircles encoding etanercept > effectiveness of MSCs alone	↓Th17 cells in spleen ↓Osteoclastogenesis
(93)	Rheumatoid arthritis	Umbilical cord stroma	None	Mice	↓Clinical score ↓Histopathology score	↓Th1 cells ↓Th17 cells ↓Serum levels of autoantibodies ↓T follicular helper (Tfh) cells ↑Treg cells ↓Pathogenic IL17^+^IFN-γ^+^ T cells ↓Pathogenic IL17^+^Foxp3^+^ T cells
(94)	Rheumatoid arthritis	Adipose tissue	None	Mice	↓Clinical score	↑IL-10 ↑TGF-β ↑IDO ↓Th17 cells ↑Treg cells ↓IFN-γ^+^CD4^+^ Th1 cells ↓IL-17^+^CD4^+^ Th17 cells
(96)	Rheumatoid arthritis	Adipose tissue	None	Mice	↓Clinical score ↓Histopathology score	↑Tregs expansion in the peripheral blood and spleen ↓TNF-α ↓IL-6 ↓IL-1β ↑IL-10 ↓Type II collagen IgG antibody ↓Type II collagen IgG2a antibody ↓Proliferation of human primary T cells
(95)	Rheumatoid arthritis	Umbilical cord stroma	None	Rats	↓Paw edema ↓Clinical score Effectiveness umbilical cord-derived MSCs > effectiveness bone marrow-derived MSCs	↓T-cell activation ↑Tregs expansion
		Bone marrow				
(88)	Rheumatoid arthritis	Placenta	None	Rats	↓Clinical score ↓Histopathology scores	↓TNF-α ↓IFN-γ ↑SOD ↑GSH-Px ↑T-AOC ↑CD4^+^/CD8^+^ T-cell ratio
(97)	Rheumatoid arthritis	Umbilical cord stroma	None	Rats	↓Clinical score ↓Prothrombotic state	↑CD4^+^CD25^+^ Tregs expansion ↑Antithrombin ↓IL-1β ↓IL-17 ↓TNF-α ↓VEGF ↓Tissue factor
(98)	Ankylosing spondylitis	Bone marrow	None	Humans	↑Proportion of patients who achieved ASAS20 ↑Proportion of patients who achieved ASAS40 ↑Mean ASAS20 duration ↓Mean Ankylosing Spondylitis Disease Activity Score ↓Mean Bath Ankylosing Spondylitis Functional Index	↓Average total inflammation extent

*Both the methodology employed and the results obtained by each article are represented in this table. IL-5, interleukin 5; Tr1 cells, type 1 regulatory T cells; CCL5, C-C motif chemokine ligand 5; CXCL2, C-X-C motif chemokine ligand 2; SOD, superoxide dismutase; GSH-Px, glutathione peroxidase; T-AOC, total antioxidant capacity; NLRP3, NACHT, LRR and PYD domains-containing protein 3; IgG2a, immunoglobulin G2a*.

#### Treatment of chronic inflammatory disorders of the intestine

Inflammatory bowel disease is a group of inflammatory conditions of the colon and small intestine. Crohn's disease and ulcerative colitis are the main types of inflammatory bowel disease (198). Crohn's disease is a granulomatous disease that can reach any part of the gastrointestinal tract, from the mouth to the anus. In the Crohn's disease, the terminal ileum and the cervix are the most frequently affected areas. The clinical presentation of the disease can range from recurrent bouts of diarrhea, fever, severe abdominal pain, weight loss and of systemic complications, drastically affecting individual's quality of life (199). Ulcerative colitis, however, is an idiopathic inflammation that specifically affects the cervix and rectum. Clinically, the ulcerative colitis is characterized by episodes of recurrent bloody diarrhea, followed by tenesmus and severe abdominal cramps. In contrast to Chron's disease, in the ulcerative colitis the ulceration does not reach the muscular layer of the mucosa and the inflammation is limited to the mucosa and the lamina propria (200). The symptoms observed in Crohn's disease result from an altered intestinal immune system response that triggers the excessive release of cytokines such as TNF-α, IFN-γ, IL-12, IL-13, and IL-17, secreted by Th1 cells. On the other hand, the IL-4 and IL-5 cytokines involved in ulcerative colitis are secreted by Th2 cells (201). The initial alteration in the mucosa and submucosa tunics arises from the infiltration of inflammatory cells in the crypts of Lieberkuhn (202). Inflammatory bowel diseases were treated with hMSCs in 21 (99–117, 155, 156) out of the 132 articles analyzed. In two studies (116, 117), bone marrow-derived hMSCs were used for the treatment of Crohn's disease in humans. In the other 19 (99–115, 155, 156) articles analyzed, hMSCs were used for the treatment of experimental colitis in animal models. Among them, 16 (99–115, 155, 156) used mice and three (108, 111, 112) used pigs as the experimental model. Regarding the source of the hMSCs used, in seven studies (100, 101, 103, 107, 113, 115, 155) hMSCs were isolated from the umbilical cord. Furthermore, hMSCs were isolated from the bone marrow (99, 102, 108, 109, 111, 112) and adipose tissue (100, 103, 104, 106, 110, 156) in six studies each. The menstrual blood (105), dental pulp (114) and gingival (99) was chosen as the source of hMSCs in only one study each. In two studies (102, 103), hMSCs were obtained from the directed differentiation of embryonic stem cells.

Among the animal studies, the cumulative survival rate, the percentage of body weight change, the disease activity index score, the histological damage score, the macroscopic damage score, the change in colon weight, the change in colon length, the change in colon weight-to-length ratio and the intra-colon myeloperoxidase activity were the outcomes used by most studies selected in this systematic review to assess the potential of hMSCs administration for the treatment of ulcerative colitis. In both the two human clinical trials selected, the change in the Crohn's disease activity index (CDAI) score was chosen as the primary endpoint that was used to assess the disease activity in Crohn's disease and to evaluate both the induction of response and the maintenance of response following hMSCs administration. However, the CDAI score is composed of some variables that are subjective and therefore vulnerable to observation bias. We therefore propose that the CDAI score should be used in combination with other endpoints that are less susceptible to bias such as the endoscopic disease activity score and the histologic disease activity score in order to correctly assess the effectiveness of hMSCs administration for the treatment of Crohn's disease in clinical trials. Treatment of inflammatory bowel diseases with hMSCs resulted in an increase in the survival rates (101–103, 108, 109, 155, 156) and in a decrease in the severity (99–117, 155, 156) of the disease, as described by many of the articles selected. Furthermore, a reduction in the pathology of the colon (99, 100, 101, 102, 103, 104, 106, 107, 108, 109, 110, 112, 114, 115, 155, 156) and a recovery in the destruction of the epithelial barrier (106) was also frequently observed. This reduction in the pathology of the colon was further confirmed in a study conducted by Arturo et al. (117), in which a decrease in the levels of both anti-*Saccharomyces cerevisiae* antibodies and antinuclear autoantibodies in Crohn's disease patients treated with hMSCs was observed 1 year after the beginning of the treatment. Additionally, a study by Robinson et al. (112) demonstrated that the administration of hMSCs reduced the damage to nerve processes in the colonic wall, protected against myenteric neuronal loss and prevented changes in neuronal subpopulations in a guinea-pig model of 2,4,6-trinitrobenzene-sulfonate-induced colitis.

In general, the studies analyzed in this systematic review demonstrated that the levels of pro-inflammatory and immunoregulatory cytokines were significantly affected by the treatment with hMSCs. In some of the studies selected, administration of hMSCs reduced the levels of serum amyloid A protein (108) and pro-inflammatory cytokines such as IFN-γ (99, 100, 102, 103, 108, 117, 155, 156), TNF-α (100, 102–104, 108, 109, 113, 116, 155, 156), IL-2 (102, 104, 117), IL-12 (103, 109, 156), IL-16 (106), IL-17 (99, 100, 106, 108, 113), IL-1β (100, 103, 106, 109, 114, 152), LIF (114), CCL5 (103, 109, 156), CCL2 (106), CXCL2 (103, 156), CXCL9 (106), and CXCL10 (106) and increased the levels of immunoregulatory cytokines such as IL-10 (99, 103, 104, 106, 108, 109, 116, 117, 155, 156) and IL-4 (104, 108). Furthermore, IL-6, a cytokine with both inflammatory and immunoregulatory properties, was found to be decreased in some studies (99, 100, 103, 108, 109, 113, 114, 155, 156) while, in others, the expression of this cytokine was stimulated by the administration of hMSCs (116, 117). The administration of hMSCs had also a stimulatory effect in the expression of TGF-β (106, 109, 117), PGE2 (101, 114, 155), PTGES (114), IDO (99, 108, 112), iNOS (99, 106), COX-2 (99, 101, 109), TNFSF14 (106), and Arg-1 (106) and an inhibitory effect in the expression of TIMP metallopeptidase inhibitor 1 (106) and in the myeloperoxidase activity (106) in the colon. The immune inhibitory ligandPD-L1 is also highly expressed by hMSCs, as demonstrated in a study conducted by Wang et al. (102).

Effects of the administration of hMSCs in the proliferation, differentiation and migration of immune cells were also analyzed by the majority of the studies selected and the results demonstrated that the use of hMSCs lead to an inhibition in the proliferation and infiltration of inflammatory cells into the colon. As reported by some studies, the administration of hMSCs inhibited the activation and stimulated the apoptosis of T lymphocytes (99, 102, 104, 109, 113, 115). The clonal expansion of both B cells (115) and CD8^+^and CD4^+^ T cells (102, 104) was also inhibited by the treatment with hMSCs. In particular, a significant inhibition in the clonal expansion of CD4^+^IL17^−^IFN^+^Th1 (103, 113) and CD4^+^IL17^+^IFN-γ^−^ Th17 (113) cells was observed following the administration of hMSCs. Additionally, an opposite effect was reported in the clonal expansion of CD4^+^CD25^+^FoxP3^+^and CD4^+^CD127^+^Treg (103, 104, 113, 156) cells after the administration of hMSCs. Furthermore, a study conducted by Lv et al. (105) reported a significant decrease in the number of CD11c^+^MHC-II^+^ dendritic cells in the spleen of hMSC-treated mice. In another study, Song et al. (107) demonstrated that the administration of extracts of hMSCs shifted the macrophage functional phenotype from M1 to M2 in the colon and peritoneum of mice with induced colitis. This shift in the functional phenotype of macrophage was conducted through the reduction in the levels of CCL2, CXCL9, and iNOS and the increase in the levels of IL-10, TNFSF14, CCL1, andArg-1 observed after the treatment with extracts of hMSCs. The effects of the presence of hMSCs in a population of macrophages were also studied by Anderson et al. (110). This study demonstrated that the presence of hMSCs induced the generation of macrophages with characteristics that are distinct from classically activated macrophages. These macrophages generated presented high arginase activity, increased secretion of IL-10 after restimulation and potent immunosuppressive activity on T cells and other macrophages. The generation of this type of macrophages appeared to be driven by the activation of COX-2 in hMSCs. Lastly, the infiltration of immune cells into the colon was also affected by the administration of hMSCs. In general, treatment with hMSCs inhibited the infiltration of leukocytes (107, 110, 111, 155) and CD4^+^ (99, 116, 155) T lymphocytes into the colon. Specifically, treatment with hMSCs inhibited the infiltration of MPO^+^ neutrophils, Mac-1^+^ macrophages, Mac-1^+^ NK cells and Mac-1^+^ granulocytes into the colon, as reported by a study conducted by González et al. (104). The infiltration of CD4^+^CD25^+^FoxP3^+^Treg cells into the colon was, however, found to be stimulated by the administration of hMSCs in the majority of the articles selected (99, 116, 155). It is, therefore, possible to hypothesize that the decrease in the pathology of the colon and the recovery in the destruction of the epithelial barrier observed following the treatment with hMSCs is a consequence of the ability of these stem cells to inhibit the proliferation and infiltration of inflammatory cells such as CD8^+^, Th1 and Th17 lymphocytes, neutrophils, B cells, NK cells, M1 macrophages and granulocytes into the colon and to stimulate the proliferation of M2 macrophages and cells with a regulatory phenotype such as Treg lymphocytes. As a result, the level of pro-inflammatory cytokines such as IFN-γ, TNF-α, IL-2, IL-12, and IL-17 also decreases, culminating in a reduction in the pathological process. In addition, the secretion of IL-6, TGF-β, PGE2, and IDO by hMSCs may be the mechanism responsible for both the shift in the macrophage functional phenotype from M1 to M2 and the inhibition in the infiltration of MPO^+^ neutrophils, Mac-1^+^ macrophages, Mac-1^+^ NK cells and Mac-1^+^ granulocytes into the colon, resulting in a decrease in the myeloperoxidase activity in the intestine, which reduces both the tissue damage and the inflammation that is needed for the occurrence of the pathological process.

Celiac disease is an autoimmune inflammatory enteropathy caused by the ingestion of gluten in genetically susceptible individuals. Ingestion of gluten by these patients results in a chronic inflammatory response in the mucosa of the small intestine, which is accompanied by atrophy of the villi and hyperplasia of the intestinal crypts. The clinical manifestations of the celiac disease are very variable, and the patients affected by the disease may be asymptomatic, manifest symptoms of intestinal malabsorption or extraintestinal manifestations such as dyspepsia, fatigue, infertility, neurological diseases, osteoporosis and dermatitis herpetiformis (203). In patients affected by the celiac disease, the passage of gliadin peptides by the intestinal submucosa through transferrin receptors CD71 acts by activating CD4^+^ T lymphocytes, which recognize these peptides through T cell receptors (204). As a result, a stimulation of a Th1 and/or Th2 type response occurs, culminating in the secretion of pro-inflammatory cytokines such as IL-15, IFN-γ, IL-17, IL-21, and IL-23, which damage the intestinal mucosa. The secretion of IFN-γ stimulates fibroblasts to secrete metalloproteinases, which act degrading the collagen, glycoproteins and proteoglycans of the extracellular matrix, resulting in villous atrophy (205). The treatment of type II refractory celiac disease was carried out with hMSCs in only one study (118), which used the bone marrow as the source of hMSCs and was conducted in humans. This study was a single case report and used the stool frequency, the change in mucosal architecture, and the percentage of body weight change as the primary endpoints that were applied in order to assess the efficacy of hMSCs administration for the treatment of celiac disease. Celiac disease, like other food and allergy-related disorders, lacks well-defined clinical endpoints. Therefore, we recommend that multiple endpoints should be combined to capture the overall activity of the disease. Possible endpoints that can be used in combination includes: intestinal permeability, histological scores, gluten concentration, villus-height-to-crypt-depth ratio and changes in the levels of biomarkers in the serum. Exploratory endpoints such as the serum levels of cytokines and the proportion of inflammatory cells in the blood can be used to assess the immunomodulatory potential of the hMSCs administration. In the study selected, a reduction in the severity of the disease was observed after the administration of hMSCs. In particular, treatment with hMSCs resulted in a full macroscopic and microscopic recovery of the gut mucosa, and, as a consequence, in the normalization in stool frequency and a decrease in the weight loss resulting from the disease. Finally, an accentuated reduction in the levels of both IL-15 cytokine and IL-15 receptor at the mucosal level were observed after hMSCs treatment. Table 5 summarizes the methodology employed and the results obtained in the studies selected in this systematic review regarding the effects of the administration of hMSCs for the treatment of chronic inflammatory disorders of the intestine.

**Table 5:** List of *in vivo* studies in which the therapeutic potential of the administration of hMSCs for the treatment of chronic inflammatory disorders of the intestine was evaluated and the results obtained.

**References**	**Autoimmune disease**	**Source of hMSC**	**Variables**	**Experimental model**	**Clinical and laboratory effects**	**Mechanism proposed**
(155)	Ulcerative colitis	Umbilical cord	None	Mice	↓Clinical score ↓Weight loss ↑Survival rates ↓Colon pathology	↑Infiltration of CD4^+^CD25^+^FoxP3^+^ Tregs in the colon ↓Infiltration of CD11b^+^ leukocytes in the colon ↓Infiltration of CD4^+^ lymphocytes in the colon ↓Proliferation of mononuclear cells ↑PGE2 ↑IL-10 ↓IL-6 ↓IFN-γ ↓TNF-α
(156)	Ulcerative colitis	Adipose tissue	None	Mice	↓Clinical score ↓Colon pathology ↓Weight loss ↑Survival rates	↓Th1-cell activation in colonic mucosa and draining lymph nodes ↑CD4^+^CD25^+^FoxP3^+^IL-10^+^ Tregs in the mesenteric lymph node ↓IL-6 ↓IL-12 ↓IL-1β ↓IFN-γ ↓TNF-α ↓CCL5 ↓CXCL2 ↑IL-10
(99)	Ulcerative colitis	Gingiva Bone marrow	None	Mice	↓Clinical score ↓Colon pathology ↓Weight loss	↓Peripheral blood lymphocyte proliferation ↑Infiltration of CD4^+^CD25^+^ FoxP3^+^ Tregs in the colon ↓Infiltration of CD4^+^ lymphocytes in the colon ↑IL-10 ↑IDO ↑iNOS ↑COX-2 ↓IL-6 ↓IL-17 ↓IFN-γ
(100)	Ulcerative colitis	Umbilical cord Adipose tissue	None	Mice	↓Clinical scores ↓Colon pathology ↓Weight loss	↓IL-6 in the serum ↓IL-17 in the serum ↓TNF-α in the serum ↓IL-1β in the serum ↓IFN-γ in the serum ↓IL-10 in the serum
(101)	Ulcerative colitis	Umbilical cord	Administration of late-passage MSCs	Mice	None	↑COX-2 ↑PGE2
			Administration of early-passage MSCs		↓Weight loss ↑Survival rates ↓Clinical score ↓Colon pathology Effectiveness early-passage MSCs > effectiveness late-passage MSCs	
(102)	Ulcerative colitis	Embryonic stem cells	None	Mice	↓Weight loss ↓Clinical score ↓Colon pathology	↓T lymphocyte proliferation ↓B lymphocyte proliferation ↑PD-L1 expression
(103)	Ulcerative colitis	Embryonic stem cellsAdipose tissueUmbilical cord	None	Mice	↑Survival rates ↓Weight loss ↓Clinical score ↓Colon pathology	↓Inflamatory cell infiltration in the colon ↓Cycling CD4 T-cells ↓Activated T cells ↓IL-2 ↓TNF-α ↓IFN-γ
(104)	Ulcerative colitis	Adipose tissue	None	Mice	↑Survival rates ↓Weight loss ↓Clinical score ↓Colon pathology	↓Intra-colonTNF-α ↓Intra-colon IFN-γ ↓Intra-colon IL-1β ↓Intra-colon IL-6 ↓IL-12 ↓Intra-colon CCL5 ↓Intra-colon CXCL2 ↑Intra-colon IL-10 ↓IFN-γ^+^Th1 cell expansion ↑CD4^+^CD25^+^FoxP3^+^ Tregs expansion
(105)	Ulcerative colitis	Menstrual blood	None	Mice	↓Disease activity index ↓Weight loss ↓Colon pathology	↓Intra-colon TNF-α ↓Intra-colon IL-2 ↑Intra-colon IL-4 ↑Intra-colon IL-10 ↓Intra-colon MPO^+^ neutrophils ↓Intra-colon Mac-1^+^ macrophages ↓Intra-colon Mac-1^+^ NK cells ↓Intra-colon Mac-1^+^ granulocytes ↓CD3^+^CD25^+^ active T cells in the spleen ↓CD3^+^CD8^+^ T cells in the spleen ↓CD11c^+^MHC-II^+^ dendritic cells in the speen ↑CD4^+^CD25^+^FoxP3^+^ Tregs expansion
(106)	Ulcerative colitis	Adipose tissue	None	Mice	↓Weight loss	↑Adipose-tissue derived MSCs in popliteal, mesenteric parathymic and parathyroid lymph nodes
(107)	Ulcerative colitis	Umbilical cord	Administration of extracts of MSCsAdministration of MSCs	Mice	↓Disease activity index ↓Weight loss ↓Colon pathology ↑Intestinal epithelial barrier Effectiveness administration of extracts of MSCs >effectiveness administration of MSCs	↓M1 macrophages ↑M2 macrophages ↓Myeloperoxidase activity in the colon ↓Intra-colon CCL2 ↓Intra-colon CXCL9 ↓Intra-colon iNOS ↓Intra-colon IL-17 ↑Intra-colon TGF-β1 ↑Intra-colon IL-10 ↑Intra-colon TNFSF14
						↑Intra-colon CCL1 ↑Intra-colon Arg-1 ↓Intra-colon sICAM-1 ↓Intra-colon IL-1β ↓Intra-colon IL-16 ↓Intra-colon CXCL10 ↓Intra-colon TIMP metallopeptidase inhibitor 1
(109)	Ulcerative colitis	Bone marrow	Stimulation of MSCs with IFN-γ prior to administrationAbsence of stimulation with IFN-γ	Mice	↓Weight loss ↑Survival rates ↓Clinical score ↓Colon pathology ↓Serum amyloid A protein Effectiveness MSCs + IFN-γ > effectiveness MSCs alone	↑IDO ↓PBMC proliferation ↓CD3^+^ T cells infiltration in the colon ↓Intra-colon TNF-α ↓Intra-colon IL-6 ↓Intra-colon IL-17 ↓Intra-colon IFN-γ ↑Intra-colon IL-4 ↑Intra-colon IL-10
(110)	Ulcerative colitis	Adipose tissue	None	Mice	↓Weight loss ↑Survival rates ↓Clinical score ↓Disease recurrence ↓Colon pathology	↑IL-10 secretion ↓IL-12 secretion ↓T cells proliferation ↓Colon neutrophil infiltration ↓Macrophages proliferation ↑Arginase activity ↑COX-2 activity ↓Intra-colon TNF-α ↓Intra-colon IL-6 ↓Intra-colon IL-1β ↓Intra-colon CCL5 ↑TGF-β1
(113)	Ulcerative colitis	Umbilical cord	None	Mice	↓Clinical score ↓Colon pathology ↓Weight loss	↓T-cell proliferation ↑IDO
(114)	Ulcerative colitis	Dental pulp Bone marrow	Stimulation of MSCs with acetylsalicylic acid prior to administrationAbsence of stimulation with acetylsalicylic acid None	Mice	↓Weight loss ↓Disease activity index ↓Histologic activity index Effectiveness MSCs treated with acetylsalicylic acid > effectiveness MSCs alone	↑AnnexinV^+^7AAD^+^ apoptotic T cells ↓CD4^+^IL17^+^IFN^−^ Th17 cells ↓CD4^+^IL17^−^IFN^+^ Th1 cells ↑CD4^+^CD25^+^Foxp3^+^ Tregs ↓TNF-α ↓IL-6 ↓IL-17
(115)	Ulcerative colitis	Umbilical cord	Stimulation of MSCs with 5-azacytidine prior to administration Absence of stimulation with 5-azacytidine	Mice	↓Weight loss ↑Survival rates ↓Disease activity index ↓Colon pathology Effectiveness MSCs treated with 5-azacytidine > effectiveness MSCs alone	↓Infiltration of lymphocytes in the colon ↓Mononuclear cells ↓Jurkat cells ↑COX2 gene ↑PTGES gene ↑PGE2 ↓LIF gene ↓IL-6 gene ↓IL-1β gene
(108)	Ulcerative colitis	Bone marrow	None	Pigs	↓Clinical score ↓Colon pathology ↓Enteric neuropathy associated with intestinal inflammation	↓CD45^+^ leucocytes infiltration in the colon ↑Nerve fiber density in the mucosa ↑Morphology of β-tubulin (III)-IR fibers in mucosal and muscular layers of colon sections ↓Myenteric neuronal loss
(111)	Ulcerative colitis	Bone marrow	None	Pigs	↓Weight loss ↓Colon pathology ↓Neuronal cell body hypertrophy in colon	↓CD45^+^ leucocytes infiltration in the colon ↓Myenteric neuronal loss
(112)	Ulcerative colitis	Bone marrow	None	Pigs	↓Weight loss ↑Repair of damaged tissue and nerve fibers	↓CD45^+^ leucocytes infiltration in the colon wall and the myenteric plexus ↓Myenteric neuronal loss
(116)	Crohn's disease	Bone marrow	None	Humans	↓Crohn's disease activity index score	↓Peripheral blood mononuclear cell proliferation ↓TNF-α ↑IL-1β ↑IL-6 ↑IL-10 ↓CD4^+^ T cells in the colon ↑CD4^+^CD127^+^ Tregs in the colon
(117)	Crohn's disease	Bone marrow	None	Humans	↓Crohn's disease activity index score	↑IL-10 ↑TGF-β ↑IL-6 ↓IFN-γ ↓IL-2 ↓Antinuclear autoantibodies ↓Anti-*Saccharomyces cerevisiae* antibodies
(118)	Type II refractory celiac disease	Bone marrow	None	Humans	↓Stool frequency ↓Weight loss ↓Gut pathology	↓IL-15 cytokine in the small intestinal mucosa ↓IL-15 receptor in the small intestinal mucosa

*Both the methodology employed and the results obtained by each article are represented in this table. iNOS, inducible nitric oxide synthase; MPO, myeloperoxidase; Mac-1, macrophage-1 antigen; TNFSF14, tumor necrosis factor superfamily member 14; CCL1, C-C motif chemokine ligand 1; Arg-1, arginase 1; sICAM-1, soluble intercellular adhesion molecule-1; IL-16, interleukin 16; CXCL10, C-X-C motif chemokine 10; PTGES, prostaglandin E synthase; LIF, leukemia inhibitory factor*.

#### Treatment of autoimmune disorders of the lungs

Idiopathic pulmonary fibrosis is a chronic, inflammatory, progressive and fibrosing disease, limited to the lungs. The disease is characterized by pulmonary interstitial fibrosis, with a radiological and/or histological pattern of usual interstitial pneumonia, with poor prognosis (206). Idiopathic pulmonary fibrosis is triggered by an alveolar lesion that leads to the activation of TGF-β and disruption of the basal alveolar membrane. In the presence of persistent lesion pathways or altered repair mechanisms, activated TGF-β may lead to alveolar apoptosis and transformation of the epithelial-mesenchymal transition, with fibroblasts and fibrocytes differentiating into apoptosis-resistant myofibroblasts (207). The resulting excessive collagen and fibronectin deposition results in a chronic fibrosing process leading to idiopathic pulmonary fibrosis. In two studies (119, 120), hMSCs were used for the treatment of autoimmune disease-associated lung fibrosis. Both studies used the mice as the experimental model and, while the adipose tissue was chosen as the source of hMSCs in one study (120), in the other study (119), hMSCs were isolated from the bone marrow and umbilical cord. The cumulative survival rate, the lung weight, the hydroxyproline levels in the lungs and the Ashcroft's modified score for lung fibrosis were the outcomes used by most studies selected in this systematic review to assess the potential of hMSCs administration for the treatment of autoimmune disease-associated lung fibrosis. Because there were no human clinical trials among the studies selected, it was not possible to identify primary endpoints commonly used to evaluate the effectiveness of hMSCs administration for the treatment of autoimmune disease-associated lung fibrosis in humans. There is not a consensus regarding what constitutes the best primary endpoint that should be used in idiopathic pulmonary fibrosis clinical trials. Therefore, we propose that multiple endpoints such as the forced vital capacity, the single breath diffusing capacity for carbon monoxide, the 6-min walk test, and the cumulative survival rate should be used in conjunction. Exploratory endpoints such as the serum levels of inflammatory cytokines and profibrotic mediators can also be used as they provide important information about the ability of hMSCs to influences the pathological course of the disease. In both studies, an increase in the survival rates and a reduction in the severity of the disease was observed in conjunction with a decrease in the lung pathology. Regarding the mechanisms proposed for the action of hMSCs, Reddy et al. (120) described that the administration of hMSCs down-regulated the expression of both pro-inflammatory cytokines such as IL-2, IL-1β, TNF-α, and TGF-β and pro-fibrotic mediators such as bFGF, CTGF, COL3a1, and CoL1a1, leading to a reduction in inflammation and pulmonary fibrosis. In this study, it was also observed a downregulation in the expression of both matrix metalloproteinases (MMP) and tissue inhibitor of metalloproteinases (TIMP), resulting in the maintenance in the MMP-TIMP balance and preventing the restructuring of the matrix following the lung injury. In addition, in a study conducted by Liu et al. (119), lower levels of pro-inflammatory cytokines IL-6, IL-8, CCL2, IFN-γ, TNF-α and higher levels of TGF-β1 and CXCL10 were found to be associated with the presence of hMSCs. Specifically, Liu et al. (119) showed that hMSCs is able toblock α-SMA activation through a TGF-β1-mediated mechanism. This study also demonstrated that hMSCs promoted CD4^+^CD25^+^CD127^(low/−)^/foxp3^+^Tregs expansion in T cell subsets from patients with idiopathic pulmonary fibrosis and inhibitedCD3^+^CD8^+^cytotoxic T cells and CD3^+^CD56^+^ NKT cells proliferation in an experimental cell model. Table 6 summarizes the methodology employed and the results obtained in the studies selected in this systematic review regarding the effects of the administration of hMSCs for the treatment of autoimmune disorders of the lungs.

**Table 6:** List of *in vivo* studies in which the therapeutic potential of the administration of hMSCs for the treatment of autoimmune disorders of the lungs was evaluated.

**References**	**Autoimmune disease**	**Source of hMSC**	**Variables**	**Experimental model**	**Clinical and laboratory effects**	**Mechanism proposed**
(119)	Idiopathic pulmonary fibrosis	Bone marrow Umbilical cord	None	Mice	↑Survival rates ↓Pulmonary inflammation and fibrosis Effectiveness bone marrow-derived MSCs > effectiveness umbilical cord-derived MSCs	↓CD3^+^CD56^+^ NKT cells ↓CD3^+^CD8^+^ T cell induction ↑CD3^+^CD4^+^ T cells ↑CD4^+^CD25^+^CD127^(low/−)^/foxp3^+^ Tregs ↓IFN-γ ↓TNF-α ↓IL-6 ↓IL-8 ↓CCL2 ↓α-SMA activation ↑TGF-β1 ↑CXCL10
(120)	Idiopathic pulmonary fibrosis	Adipose tissue	None	Mice	↑Survival rates ↓Ashcroft's modified score for lung fibrosis ↓Lung weight ↓Lung pathology ↓Collagen deposition in the lungs	↓IL-2 ↓IL-1β ↓TNF-α ↓TGF-β ↓bFGF ↓CTGF ↓COL3a1 ↓CoL1a1 ↓Matrix metalloproteinases ↓Tissue inhibitor of metalloproteinases

*Both the methodology employed and the results obtained by each article are represented in this table. α-SMA, alpha-smooth muscle actin; IL-8, interleukin 8; CTGF, connective tissue growth factor; COL3a1, collagen alpha-1(III) chain; CoL1a1, collagen, type I, alpha 1*.

#### Treatment of autoimmune neurologic disorders

The treatment of autoimmune neurologic disorders was conducted with hMSCs in 31 (102, 121–149, 154) out of the 132 studies (33–164) selected. Among them, in 27 studies (102, 121–145, 154) hMSCs were applied for the treatment of multiple sclerosis while in three studies (146–148) they were used for the treatment of autoimmune myasthenia gravis and in only one study (149) hMSCs were used for the treatment of neuromyelitisoptica.

Multiple sclerosis is a demyelinating disease of the central nervous system of an inflammatory, chronic and progressive nature. The destruction of the myelin sheath and axonal degeneration results in scattered lesions in the central nervous system, especially in the optic nerves, brainstem, spinal cord and periventricular white matter. The dissemination of these lesions results in neurological deficits of variable course (208). The infiltration of activated T lymphocytes and the secretion of inflammatory mediators by these cells results in endothelial changes in the blood-brain barrier and stimulate the inflammatory cascade (209). The production of IFN-γ by activated TH1 lymphocytes activates macrophages that secrete proteases and TNF-α, which contribute to the destruction of oligodendrocytes. The activation of macrophages by IFN-γ also results in the production of high levels of nitric oxide. This increase in nitric oxide inhibits mitochondrial respiration and reduces the synthesis of ATP, leading to the axonal injury observed in the pathological process of the disease (210). Regarding the studies in which hMSCs were used for the treatment of multiple sclerosis (102, 121–145, 154), five studies (125, 131, 132, 134, 135) were conducted in humans, 19 (102, 121–124, 126–129, 133, 136–139, 141, 143–145, 154) were conducted in mice and three studies (130, 140, 142) used rats as the experimental model. Among these studies, 16 used hMSCs isolated from the bone marrow (102, 122, 123, 125–128, 131–137, 138, 143, 154), seven isolated hMSCs from the adipose tissue (121, 128, 130, 136, 140, 141, 145), four isolated hMSCs from the umbilical cord (124, 128, 142, 144) and two isolated hMSCs from the placenta (129, 139). In only one study (102), hMSCs were obtained from the differentiation of embryonic stem cells.

Among the animal studies, the cumulative survival rate, clinical score, disease incidence, disease onset, number of infiltrating cells, inflammation area, degree of demyelination, degree of axonal damage, death of oligodendrocytes, glial activation, number of astrocytes, total lesion area and the total distance traveled, average velocity and time spent in motion over 5 min were the outcomes used by most studies selected in this systematic review to assess the potential of hMSCs administration for the treatment of multiple sclerosis. In the human clinical trials selected, the change in the expanded disability status scale (EDSS) score was chosen as the primary endpoint that was used to assess the stabilization or improvement of general progression of the disease following hMSCs administration. However, there are many limitations associated with the used of this score. First of all, due to the subjective nature of the neurological examination, the EDSS score is composed of some variables that are subjective and therefore susceptible to observation bias. Second, the EDSS score is not appropriate to assess the rate of progression of multiple sclerosis as this score is not linear. Lastly, several aspects of the progression of the disease are not sufficiently assessed by this score. Therefore, we propose that the EDSS score should be used in conjunction with secondary endpoints that are less susceptible to bias, reflect the progression or remission of the disease and are able to assess the aspects of the progression of the disease that are not evaluated by the EDSS score. In this regard, secondary endpoints such as the size and number of plaques, the cumulative number of T2-hyperintense and gadolinium-enhancing T1 lesions, gray and white matter volume, the percentage of brain volume change, and the number of relapses should be used in conjunction with the EDSS score in order to assess the effectiveness of hMSCs administration for the treatment of multiple sclerosis in clinical trials. Exploratory endpoints such as the T and B cell population frequency in blood and the serum level of cytokines can be applied to assess additional immunomodulatory effects of the hMSCs infusion. In the majority of the studies selected the administration of hMSCs reduced the severity and clinical parameters and delayed the progression of the disease (102, 121–126, 128–139, 141–145, 154). This improvement in the clinical condition was usually accompanied by an increase in the myelin levels (121, 136, 137) and a reduction in the number of apoptotic cells (142), in vascular congestion (130), in axonal injury (124) and in the extent of the chronic demyelinated regions in the central nervous system and spinal cord (122, 124, 126, 133, 142, 154). Furthermore, an increase in the number of oligodendrocyte lineage cells (122) and NeuN-positive neurons in the gray matter and spinal cord (142) following hMSCs administration was also observed. A decrease in the number of microglia cells (142), a reduction in astrogliosis (122, 140), an inhibition in the activation of glial cells in lesion areas (126) and a decrease in the blood-brain barrier permeability (133) was also found to be associated with hMSCs administration.

In general, the administration of hMSCs reduced the levels of pro-inflammatory cytokines such as IFN-γ (122, 126, 128, 130, 133, 141–144), TNF-α (122, 124, 126, 128, 133, 142, 154), IL-1 (124), IL-2 (122, 128), IL-12 (122), and IL-17 (121, 122, 124, 128, 130, 138, 143, 144, 154) and increased the levels of anti-inflammatory cytokines such as IL-4 (122, 124, 126, 130, 133, 138, 140, 142, 143, 144), IL-5 (122), and IL-10 (124, 126, 128, 130, 133, 138, 139, 141–143). The level of the pro and anti-inflammatory cytokine IL-6 was found to be increased in some studies (128, 136) while in others the level of this cytokine decreased (124, 154) after the administration of hMSCs. In addition, some of the studies selected suggested that the immunomodulatory properties of hMSCs can be partially attributed to the expression of molecules with immunomodulatory functions such as LIF (136, 141), PDL-1 (102, 141), COX-2 (141), TGF-β1 (141, 143), TSG-6 (141), CD200 (141), HGF (141, 143), IDO-1 (139, 141), VEGF (143), HLA-G (130, 141), HLA-E (141), and HO-1 (141) by these cells. Furthermore, a study conducted by Tafreshi et al. (141) reported that neurotrophic factors such as BDNF, CNTF, GDNF, NGF, and NTF3 are also constitutively expressed by hMSCs, evidencing the potential of this type of stem cell for paracrine support in a neurodegenerative setting. Finally, a study conducted by Hou et al. (143) also demonstrated that hMSCs administration acted by inhibiting matrix metalloproteinase 2 and 9 activities in the spinal cord of mice with experimental autoimmune encephalomyelitis.

The proliferation and differentiation of immune cells was also significantly affected by the administration of hMSCs. In general, treatment of multiple sclerosis with hMSCs resulted in an inhibition in the proliferation of both B (102) and T (102, 131, 136, 137, 141, 145) cells and in a reduction in the infiltration of inflammatory cells into the central nervous system and spinal cord (124, 126, 130, 133, 136–139, 143, 154). Specifically, administration of hMSCs led to an inhibition in the clonal expansion and infiltration of CD4^+^IFN-γ^+^ Th1 (102, 122) and CD4^+^IL-17^+^ Th17 (102, 122, 124, 138) cells into the central nervous system. Furthermore, treatment with hMSCs resulted in a stimulation in the clonal expansion of CD4^+^IL-4^+^ Th2 (122) and CD4^+^CD25^+^FOXP3^+^ Treg (124, 143) cells, as reported by some studies. On the other hand, studies conducted by Llufriu et al. (134) and Guo et al. (154) reported an increase in the numbers of CD19^+^IL-10^+^ and CD19^+^CD5^+^CD1d^high^ Breg cells after the administration of hMSCs. The proliferation of both CD44^+^ and CD45RO^+^memory T-lymphocytes was also found to be reduced after the administration of hMSCs, in studies conducted by Strong et al. (136) and Zafranskaya et al. (131), respectively. Cells of the innate immune system were also affected by the treatment with hMSCs, as described by some of the studies selected. For instance, a study by Bravo et al. (139) reported a reduction in the percentages of both CD11b^+^Ly6G^+^ neutrophils and CD11b^+^Ly6C^+^ inflammatory monocytes in infiltrates of mice with experimental autoimmune encephalomyelitis treated with hMSCs. In this study, lower RORγT and higher GATA-3 transcription factor expression levels in CD4^+^ cells were also detected in experimental autoimmune encephalomyelitis mice treated with hMSCs, which suggests that the Th17 phenotype is restrained while the Th2 subset is favored by the treatment with hMSCs. In addition, a study by Donders et al. (142) reported an inhibition in the differentiation and maturation of dendritic cells in rats with experimental autoimmune encephalomyelitis treated with hMSCs, reducing antigen presentation and, as a consequence, T-cell priming. Finally, a study by Tafreshi et al. (141) demonstrated that the loss of phosphorylated GSK3β, an enzyme known for their ability to control neuroinflammation, can be recovered in neurons of experimental autoimmune encephalomyelitis mice treated with hMSCs.

**Figure 7:**
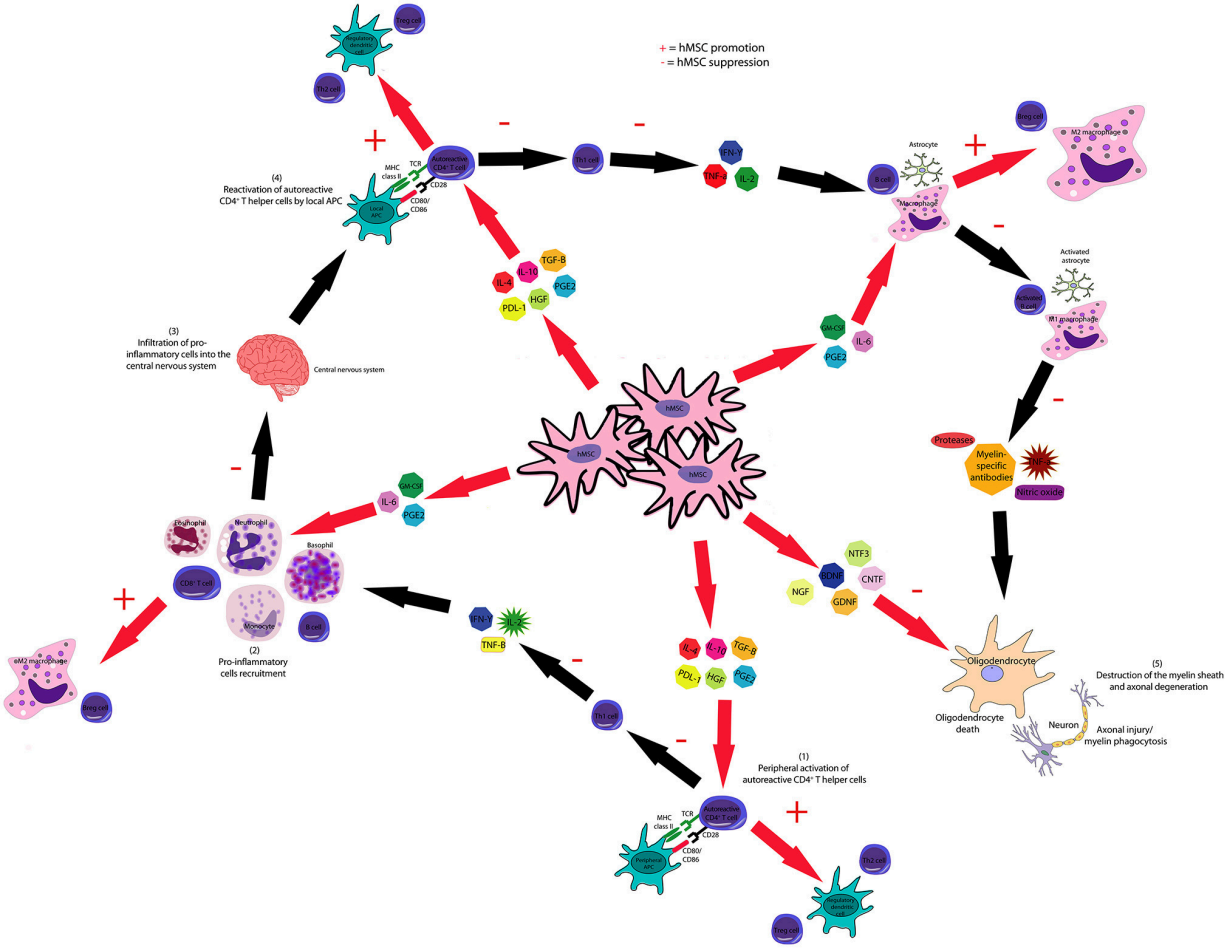
hMSCs inhibits the pathological course of multiple sclerosis through several mechanisms. hMSC-produced IL-4, IL-10, HGF, PGE2, PDL-1, and TGF-β inhibit the activation of autoreactive CD4^+^T cells and their differentiation to Th1 cells and stimulate the generation of Breg, Treg, and Th2 lymphocytes. hMSCs inhibit the activation of dendritic cells and stimulate the generation of regulatory dendritic cells. hMSC-produced IL-6, PGE2, and GM-CSF suppress astrocyte activation and macrophage polarization to M1, though favors M2 polarization. hMSCs-produced neurotrophic factors BDNF, CNTF, GDNF, NGF, and NTF3 inhibit the destruction of the myelin sheath and axonal degeneration.

Together, these results indicate that the improvement in the clinical condition observed after the administration of hMSCs is a consequence of the inhibition in the proliferation and infiltration of inflammatory cells such as B, Th1 and Th17 lymphocytes, neutrophils and monocytes into the central nervous system and spinal cord promoted by these stem cells. It can be hypothesized that factors secreted by the hMSCs administered such as IL-10, IL-4, TGF-β, PGE2, HGF, and PDL-1 acts by inhibiting the activation of autoreactive CD4^+^ T cells and their differentiation in Th1 cells in both the periphery and central nervous system and by stimulating the differentiation of these CD4^+^ T cells into Th2 and Treg lymphocytes. Additionally, these factors can stimulate APCs located in both the periphery and central nervous system to differentiate into regulatory dendritic cells, further inhibiting the activation of autoreactive CD4^+^ T cells. The recruitment of pro-inflammatory cells such as neutrophils, basophils, monocytes, eosinophils and CD8^+^T lymphocytes and their infiltration into the central nervous system can also be inhibited as a result of the decrease in the secretion of pro-inflammatory cytokines and growth factors such as IFN-γ, IL-2, and TGF-β by Th1 lymphocytes and due to the secretion of anti-inflammatory factors such as GM-CSF, PGE2, and IL-6 by the hMSCs administered. The decrease in the levels of pro-inflammatory cytokines and growth factors and the secretion of anti-inflammatory factors by hMSCs can also inhibit the activation of astrocytes in the central nervous system and stimulate pro-inflammatory cells to differentiate into M2 macrophages and cells with immunomodulatory properties such and Breg cells. As a result, the secretion of TNF-α, proteases, nitric oxide and myelin-specific antibodies by pro-inflammatory cells located in the central nervous system is also inhibited, decreasing the death of oligodendrocytes and the destruction of the myelin sheath in axons. Finally, the secretion of neurotrophic factors such as BDNF, CNTF, GDNF, NGF, and NTF3 by the hMSCs administered may also act on oligodendrocytes and neurons, further inhibiting the progression of the disease. The mechanism proposed by this systematic review concerning the inhibition in the progression of the pathological process of multiple sclerosis mediated by hMSCs is represented in Figure 7.

Myasthenia gravis is an autoimmune disorder that affects the myoneural junction, resulting in weakness and fatigability of the striated skeletal muscles. Major manifestations of the disease include diploplia, ptosis, bulbar symptoms such as weakness of the muscles of the face and throat, and generalized weakness (211). The clinical manifestations of the disease results from the production of autoreactive T cells and from the secretion of IgG autoantibodies by hyperstimulated B cells. The biding of these antibodies to nicotinic acetylcholine receptors located on the skeletal muscle membrane leads to the blockade of these receptors, increases their degradation and stimulates the complement-mediated destruction of the post-synaptic cleft, compromising the neuromuscular transmission (212). In addition, the loss of the immunosuppressive activity of Treg cells, which have a decreased expression of the FoxP3 transcription factor, results in impairment of the process of immune self-tolerance and homeostasis of the immune system (213). Two (146, 147) of the three studies (146–148) in which hMSCs were used for the treatment of autoimmune myasthenia gravis were carried out in mice and only one (148) was conducted in humans. Among them, the bone marrow was chosen as the source of hMSCs in two studies (146, 148) while in one study (147) hMSCs were isolated from the dental pulp. Among the studies conducted in mice, the change in body weight, the clinical score, the inverted screen hang time, the C3 deposit level, the neuromuscular junction IgG deposit level and the serum level of both anti-acetylcholine receptor and anti-muscle-specific tyrosine kinase antibodies were the outcomes used by most studies selected in this systematic review to assess the potential of hMSCs administration for the treatment of myasthenia gravis. In the human clinical trial selected, the change in the quantitative myasthenia gravis score (QMGC) was chosen as the primary endpoint that was used to quantify the severity of the disease following hMSCs administration. It is recommended by the Myasthenia Gravis Foundation of America that the QMGS should be used in all prospective clinical trials on myasthenia gravis (214). Currently, the QMGC is composed of 13 variables that are likely to not be affected by bias. However, due to the lack of weighting of different domains, the QMGS is, sometimes, not fully representative of myasthenia gravis severity. Therefore, other clinical and laboratory parameters should be used in conjunction with QMGS to assess the efficacy of the use of hMSCs for the treatment of myasthenia gravis in clinical trials. In addition, exploratory endpoints such as the serum level of cytokines and the T and B cell population frequency in blood can be used to evaluate additional immunomodulatory effects of the hMSCs administration.

Results from the administration of hMSCs included a reduction in the severity and clinical manifestations of the disease (146–148). Furthermore, a study conducted by Ulusoy et al. (147) demonstrated that the administration of hMSCs reduced the incidence of experimental autoimmune myasthenia gravis. In this study, treatment with hMSCs also resulted in a decrease in the levels of the pro-inflammatory cytokines IL-6 and IL-12 and inhibited the proliferation of CD11b^+^ leukocytes in the lymph nodes. The proliferation of mononuclear cells is also inhibited by the presence of hMSCs, as described by Yu et al. (146). The levels of autoantibodies that are important for the pathogenesis autoimmune myasthenia gravis were also found to be decreased following the treatment with hMSCs in all articles selected. For instance, studies conducted by Gabr and Elkheir (148) and by Yu et al. (146) reported a decrease in the levels of the anti-acetylcholine receptor antibody in the serum of patients treated with hMSCs. In particular, Yu et al. (146) also reported an inhibition in the proliferation of acetylcholine receptor-specific lymphocytes following the treatment with hMSCs. Finally, a study conducted by Ulusoy et al. (147) reported a decrease in the serum levels of anti-muscle-specific tyrosine kinase antibodies after the treatment with hMSCs. Likewise, this study described a reduction in the percentages of neuromuscular junction IgG in the serum and complement component three deposits in the muscles of mice treated with hMSCs. It can be, therefore, speculated that the reduction in the severity and clinical manifestations of the disease observed after hMSCs administration is a direct result of the inhibitory effect of these stem cells in activation and proliferation of B cells. As a consequence, the secretion of IgG autoantibodies such as the anti-acetylcholine receptor antibody and the anti-muscle-specific tyrosine kinase antibody by hyperstimulated B-cells also decreases, culminating in the inhibition in the progression of the disease.

Neuromyelitis optica is an inflammatory, demyelinating, and autoimmune disease of the central nervous system, which selectively affects the spinal cord and optic nerves, simultaneously or sequentially. Symptoms of neuromyelitis optica include loss of vision, sensitivity changes, muscle weakness, spasticity, incoordination, ataxia, urinary and fecal incontinence, and autonomic dysfunctions in parts of the trunk and limbs supplied by nerves coming out of the spine below the spinal lesion (215). Clinical and serological evidence of autoimmunity associated with B cells has been observed in patients with neuromyelitis optica, in whom demyelinating lesions exhibit perivascular immunoglobulin deposition, local activation of the complement cascade and eosinophilic infiltration (216). Other mechanisms involved in this humoral response are the secretion of IL-2, anti-myelin autoantibodies, oligodendrocyte-associated anti-glycoprotein autoantibodies, and IgG autoantibodies against the astroglial water channel aquaporin-4 (217). In general, neuromyelitis optica attacks are more severe than those of multiple sclerosis and are commonly fatal (215). The treatment of neuromyelitisoptica was carried out with hMSCs in only one study (149). This study was conducted in humans and used bone marrow-derived hMSCs for the treatment of the autoimmune disease. The study selected in this systematic review was a single case report and used the healing of pressure ulcers, the improvement of disability, the ability to walk, and the occurrence of relapse and adverse events as the primary endpoints that were used in order to assess the efficacy of hMSCs administration for the treatment of neuromyelitis optica. Because the occurrence of attacks is the main cause of neuromyelitis optica-related disability (215), we propose that the frequency and severity of attacks should be considered as the most appropriate primary endpoints in neuromyelitis optica clinical trials. In addition, exploratory endpoints such the serum levels of autoantibodies and inflammatory cytokines can be used to determinate what are the mechanisms used by the hMSCs administered to inhibit the occurrence of the pathological process. In the study selected, a reduction in both the severity and in the clinical parameters of the disease was observed following the administration of hMSCs. Table 7 summarizes the methodology employed and the results obtained in the studies selected in this systematic review regarding the effects of the administration of hMSCs for the treatment of autoimmune neurologic disorders.

**Table 7:** List of *in vivo* studies in which the therapeutic potential of the administration of hMSCs for the treatment of autoimmune neurologic disorders was evaluated.

**References**	**Autoimmune disease**	**Source of hMSC**	**Variables**	**Experimental model**	**Clinical and laboratory effects**	**Mechanism proposed**
(102)	Multiple sclerosis	Embryonic stem cells Bone marrow	None	Mice	↓Clinical score ↓Disease incidence ↑Motor functions Effectiveness embryonic stem cells-derived MSCs > effectiveness bone marrow-derived MSCs	↓IFN-γ^+^CD4^+^ Th1 cells into the central nervous system ↓IL-17^+^CD4^+^ Th17 cells into the central nervous system ↓T lymphocyte proliferation ↓B lymphocyte proliferation ↑PD-L1 expression
(121)	Multiple sclerosis	Adipose tissue	Administration of MSCs from older donors	Mice	None	
			Administration of MSCs from younger donors		↓Clinical score ↑Activity and utilization of the arena space ↑Distance traveled ↑Moving velocity ↑Myelin levels Effectiveness MSCs from younger donors > effectiveness MSCs from older donors	↓Infiltrating cells in the spinal cord ↓Splenocyte proliferation ↑IL-17 in the serum ↑IL-12 in the serum ↑IFN-γ in the serum
(122)	Multiple sclerosis	Bone marrow	None	Mice	↓Clinical score ↓Disease progression	↑Oligodendrocyte lineage cells in lesion areas ↓IFN-γ^+^CD4^+^ Th1 cells ↓IL-17^+^CD4^+^ Th17 cells ↑IL-4^+^Th2 cells ↓Infiltrating cells in the central nervous system ↓Extent of the chronic demyelinated regions ↑Oligodendrogenesis ↓Astrogliosis ↓Myelin-specific memory splenocytes ↓IFN-γ ↓TNF-α ↓IL-17 ↓IL-2 ↓IL-12 ↑IL-4 ↑IL-5
(154)	Multiple sclerosis	Bone marrow	None	Mice	↓Clinical scores ↓Demyelination in the spinal cord	↓Infiltration of inflammatory cells ↓IL-6 in the serum ↓TNF-α in the serum ↓IL-17 in the serum ↓Splenic cell production and secretion of IL-6, TNF-α and IL-17 ↑Splenic production of IL-10 ↓Splenic Th17 cells ↑CD19^+^CD5^+^CD1d^high^ Breg cells
(123)	Multiple sclerosis	Bone marrow	None	Mice	↓Disease progression ↑Survival rates ↑Motor function	↓Spleen cells proliferation
(124)	Multiple sclerosis	Umbilical cord stroma	None	Mice	↓Histopathologic scores ↓Clinical score ↓Demyelination in the spinal cord ↓Axonal injury in the spinal cord	↓Perivascular immune cell infiltration ↓IL-17^+^CD4^+^ Th17 cells ↓IFN-γ/IL-4 ratio ↑CD4^+^CD25^+^FOXP3^+^ Treg cells ↑IL-4 ↑IL-10 ↓IL-17 ↓TNF-α ↓IL-1 in the spinal cord ↓IL-6 in the spinal cord
(126)	Multiple sclerosis	Bone marrow	None	Mice	↓Average clinical score ↓Maximum clinical score ↓Demyelination of the spinal cord	↓Inflammatory cell infiltration into the central nervous system ↓Inflammatory mononuclear cell infiltration in the white matter of the spinal cord ↓CD4^+^ cells in the spinal cord ↓Activation of glial cells ↓IFN-γ in the serum ↓TNF-α in the serum ↑IL-4 in the serum ↑IL-10 in the serum ↓MMP-2 activity in the spinal cord ↓MMP-9 activity in the spinal cord
(127)	Multiple sclerosis	Bone marrow	None	Mice	None	None
(128)	Multiple sclerosis	Bone marrow	None	Mice	↓Daily mean clinical score ↓Daily mean clinical score ↓Maximal disease score ↓Cumulative disease score	↓Proliferation of mitogen or antigen-stimulated T-cells ↓IFN- γ ↓IL-2
		Adipose tissue			Effectiveness adipose tissue and umbilical cord-derived MSCs > effectiveness bone marrow-derived MSCs	↓IL-17 (Ad-MSCs and BMSCs only) ↓TNF-α (Ad-MSCs and UC-MSCs only) ↑IL-6 (BMSCs only)
		Umbilical cord stroma			↓Daily mean clinical score ↓Maximal disease score ↓Cumulative disease score	↑IL-10 (BMSCs only)
(129)	Multiple sclerosis	Placenta	Intramuscular implantation of MSCs	Mice	↓Disease progression	None
			Direct injection of MSCs into the central nervous system		↓Clinical score	
(133)	Multiple sclerosis	Bone marrow	None	Mice	↓Disease progression ↓Average clinical score ↓Maximum clinical score ↓Demyelination of the lumbar spinal cord ↓Blood-brain barrier permeability	↓Immune cell infiltration in the lumbar spinal cord ↓IFN-γ in the serum ↓TNF-α in the serum ↑IL-4 in the serum ↑IL-10 in the serum
(136)	Multiple sclerosis	Adipose tissue	Administration of MSCs isolated from lean subjects	Mice	↓Disease progression ↓Clinical score ↑Total distance traveled ↑Moving duration ↑Total velocity ↑Myelin content in the central nervous system Effectiveness MSCs from lean subjects > effectiveness MSCs from obese subjects	↓Cell infiltration into the central nervous system ↓Proliferation of T cells ↓Proliferation of memory CD44^+^ T cells
			Administration of MSCs isolated from obese subjects		↓Myelin content in the central nervous system	↑Cell infiltration into the central nervous system ↓Overall size of the lymph nodes ↑Size of the spleen ↑Proliferation of T cells ↑Differentiation of T cells into mature CD4^+^ and CD8^+^ T cells ↑IL-1 gene expression (MSCs from lean subjects) ↑IL-6 gene expression (MSCs from lean subjects) ↑IL-12 gene expression (MSCs from lean subjects) ↑PDGF gene expression (MSCs from lean subjects) ↑TNF-α gene expression (MSCs from lean subjects) ↑LIF gene expression (MSCs from lean subjects) ↑ICAM-1 gene expression (MSCs from lean subjects) ↑G-CSF gene expression (MSCs from lean subjects)
(137)	Multiple sclerosis	Bone marrow	None	Mice	↓Disease progression	↓T lymphocytes proliferation
(138)	Multiple sclerosis	Bone marrow	Administration of PSGL-1/SLeX mRNA-transfected MSCsAdministration of MSCs alone	Mice	↓Clinical score ↑Neurological function ↑ Myelination Effectiveness PSGL-1/SLeX mRNA-transfected MSCs > effectiveness MSCs alone	↑Percentage of rolling and adherent cells ↓Proliferation of CD4^+^ T lymphocytes ↓Lymphocytes infiltration into the white matter of the spinal cord
(139)	Multiple sclerosis	Placenta	None	Mice	↓Disease progression ↓Clinical score	↓Inflammatory cell infiltration ↓CD4^+^IL17^+^ Th17 cells ↓CD11b^+^Ly6G^+^ neutrophils ↓CD11b^+^Ly6C^+^ inflammatory monocytes ↓Spleen cells proliferation ↓IL-17 ↑IL-4 ↑IL-10 ↓RORγT gene ↑GATA-3 gene
(141)	Multiple sclerosis	Adipose tissue	None	Mice	↓Astrogliosis	↑GSK3β^+^ neurons in the spinal cord ↑IL-4
(143)	Multiple sclerosis	Bone marrow	None	Mice	↓Clinical score ↓Demyelination in the spinal cord ↓Astrocytes Treatment with hBM-MSCs and minocycline > treatment with hBM-MSCs or minocycline alone	↓Microglia cells ↑NeuN-positive neurons in the gray matter and spinal cord ↓Apoptotic cells ↓IFN-γ ↓TNF-α ↑IL-4 ↑IL-10
(144)	Multiple sclerosis	Umbilical cord stroma	Administration of umbilical cord derived-MSCs previously treated with IFN-γAbsence of stimulation of MSCs with IFN-γ prior to administration	Mice	↓Clinical scores ↓Disease progression Effectiveness MSCs + IFN-γ > effectiveness MSCs alone	↓Leukocyte infiltration in the spinal cord white matter ↑CD4^+^CD25^+^Foxp3^+^CD127^low/neg^ Treg cells in cervical lymph nodes cells and splenocytes ↓PBMCs proliferation (*in vitro*) ↑IL-10 ↑IL-4 ↑HGF ↑VEGF ↑TGF-β ↓IFN-γ ↓IL-17
(145)	Multiple sclerosis	Adipose tissue	None	Mice	↓Clinical scores ↑Survival rates	↓Peripheral MOG-specific T-cell responses ↓IFN-γ ↓IL-17A ↓IL-6 ↑IL-4
(130)	Multiple sclerosis	Adipose tissue	None	Rats	↓Disease progression ↓Mean clinical scores ↓Histopathologic score ↓Vascular congestion ↓Axonal loss of the gray and white matters of cerebral cortex	↓Immune cell infiltration ↑HLA-G gene expression in lymph nodes and brains ↓CD3 mRNA expression expression in the brain ↓CD19 mRNA expression expression in the brain ↓CD11b mRNA expression expression in the brain ↓IFN-γ in the serum ↓IL-17 in the serum ↑IL-4 in the serum ↑IL-10 in the serum
(140)	Multiple sclerosis	Adipose tissue	None	Rats	↓Duration of paralysis ↓Disease progression ↓Clinical score	↓Inflammatory cell infiltration in the spinal cord ↑IL-10 gene expression in the lymph node ↑IDO gene expression in the lymph node ↑IFN-γ gene expression in the lymph node
(142)	Multiple sclerosis	Umbilical cord stroma	None	Rats	↓Clinical score	↓Proliferation of activated T cells ↓Autoantigen-induced T-cell proliferation ↓Dendritic cell differentiation and maturation ↑IDO-1 ↓IFN-γ ↑IL-10 ↑LIF gene ↑PD-L1 gene ↑COX-2 gene ↑TGF- β1 gene ↑TSG-6 gene ↑CD200 gene ↑HGF gene ↑HLA-E gene ↑HLA-G gene ↑HO-1 gene ↑BDNF gene ↑CNTF gene ↑GDNF gene ↑NGF gene ↑NTF3 gene
(125)	Multiple sclerosis	Bone marrow	None	Humans	↓Expanded disability status scale score	None
(131)	Multiple sclerosis	Bone marrow	None	Humans	↓Expanded disability status scale score	↓CD45RO^+^ memory T-lymphocytes ↓Myelin-stimulated T cells proliferation
(132)	Multiple sclerosis	Bone marrow	None	Humans	↓Expanded disability status scale score	None
(134)	Multiple sclerosis	Bone marrow	None	Humans	↓Mean cumulative number of gadolinium-enhancing lesions ↓Expanded disability status scale score	↑ CD19^+^IL-10^+^ Breg cells frequency ↓Breg/CD19^+^ cells ratio ↓Th1/Th17 cells ratio
(135)	Multiple sclerosis	Bone marrow	None	Humans	↓Expanded disability status scale score ↑Sensory function ↑Pyramidal function ↑Cerebellar function	None
(146)	Autoimmune myasthenia gravis	Bone marrow	None	Mice	↓Mean clinical score	↓Anti-acetylcholine receptor antibody levels (in the serum) ↓AchR-specific lymphocyte proliferation (in the serum) ↓Mononuclear cells proliferation
(147)	Autoimmune myasthenia gravis	Dental pulp	None	Mice	↓Clinical score ↑Inverted screen hang time ↓Disease incidence ↓Complement component three deposits in the muscle	↓Anti-muscle-specific tyrosine kinase antibody levels (in the serum) ↓Neuromuscular junction IgG levels (in the serum) ↓CD11b^+^ leukocytes in lymph nodes ↓IL-6 ↓IL-12
(148)	Autoimmune myasthenia gravis	Bone marrow	None	Humans	↓Quantitative myasthenia gravis score	↓Anti-acetylcholine receptor antibody levels (in the serum)
(149)	Neuromyelitis optica	Bone marrow	None	Humans	↑Healing of pressure ulcers ↑Improvement of disability ↑Ability to walk	None

*Both the methodology employed and the results obtained by each article are represented in this table. MMP-2, matrix metalloproteinase-2; MMP-9, matrix metalloproteinase-9; Ad-MSCs, adipose tissue-derived mesenchymal stem cells; Uc-MSCs, umbilical cord-derived mesenchymal stem cells; BMSCs, bone marrow-derived mesenchymal stem cells; PDGF, platelet-derived growth factor; G-CSF, granulocyte-colony stimulating factor; RORγT, RAR-related orphan receptor gamma T; GSK3β, glycogen synthase kinase 3 beta; TSG-6, tumor necrosis factor-inducible gene 6 protein; HLA-E, human leukocyte antigen E; HLA-G, human leukocyte antigen G; HO-1, heme oxygenase 1; BDNF, brain-derived neurotrophic factor; CNTF, Ciliary neurotrophic factor; GDNF, glial cell-derived neurotrophic factor; NTF3, neurotrophin-3; MOG, myelin oligodendrocyte glycoprotein; AchR, acetylcholine receptor*.

#### Treatment of autoimmune visual and auditory disorders

Uveitis corresponds to inflammation of the uvea, the middle vascular layer of the eye, and can be classified as anterior when it attacks the iris, intermediate when it affects the ciliary and vitreous body and posterior when it affects the vitreous, retina, choroid and sclera. Uveitis with involvement of more than one uveal portion is called diffuse, usually presenting bilateral involvement (218). Experimental autoimmune uveitis is an autoimmune disease mediated by organ-specific T cells and characterized by inflammation and subsequent destruction of the neural retina and adjacent tissues. Experimental autoimmune uveitis can be induced in susceptible primates and rodents after immunization with retinal autoantigens, such as the retinoid or S antigen-related interphotoreceptor protein, or through the transfer of T cells specific for these antigens (219). The treatment of autoimmune visual disorders was conducted with hMSCs in three studies (81, 150, 163), which employed a mouse model of experimental autoimmune uveitis. Among these three studies, two isolated hMSCs from the bone marrow (150, 163) and in one study (81) hMSCs were obtained from the differentiation of embryonic stem cells. The histological disease score, fundoscopic score, and the cumulative survival rate of corneal grafts were the outcomes used by most studies selected in this systematic review to assess the potential of hMSCs administration for the treatment of autoimmune visual disorders. Due to the fact that there were no human clinical trials among the studies selected, it was not possible to identify primary endpoints commonly used to evaluate the effectiveness of hMSCs administration for the treatment of autoimmune visual disorders in humans. An ideal primary endpoint should be able to both reflect the experience of the disease by the patient and capture the overall disease activity. In this regard, variables such as the disease activity score, the disease damage score and the visual acuity should be used in combination with reports of the patients regarding their visual function and quality of life obtained after the treatment with hMSCs. In addition, exploratory endpoints such as the serum levels of inflammatory cytokines and the proportion of inflammatory cells in the blood can be used to identify what are the mechanisms employed by hMSCs that are responsible for the inhibition in the occurrence of the pathological process of the disease. According to these studies, the administration of hMSCs reduced the severity (163), clinical parameters (81) and the incidence (150) of the disease. Treatment with hMSCs was also found to be associated with a reduction in the levels of the pro-inflammatory cytokine IL-12 (81). In addition, hMSCs administration inhibited the proliferation of T cells (81, 163). Specifically, treatment with hMSCs inhibited the proliferation andstimulated the apoptosis of CD4^+^ T cells (163). However, while the differentiation of CD4^+^IFN-γ^+^ Th1 (163) and CD4^+^IL-17^+^ Th17 (163) cells from naive CD4^+^ T cells was inhibited by the administration of hMSCs, the proliferation of CD4^+^CD25^+^FoxP3^+^Treg cells (81) was stimulated by the treatment with hMSCs. hMSCs administration was also found to be associated with an inhibition in the up-regulation of CD83 in dendritic cells (81). Stimulation in the proliferation of MHC class II^lo^Ly6G-Ly6C^hi^CD11b^+^ cells in draining lymph nodes was observed in a study conducted by Lee et al. (163). Additionally, these MHC class II^lo^Ly6G-Ly6C^hi^CD11b^+^ cells suppressed CD4^+^ cell proliferation, inhibited Th1 and Th17 cell differentiation and induced CD4^+^ cell apoptosis when used for the treatment experimental autoimmune uveitis in a mouse model. Finally, a study conducted by Ko et al. (150) demonstrated that lung monocytes and macrophages primed by hMSCs expressed high levels of MHC class II, B220, CD11b, and IL-10, and showed T-cell–suppressive activities independently of CD4^+^FoxP3^+^Treg cells.

Autoimmune hearing loss is characterized by the presence of sensorineural, fluctuating, usually bilateral and asymmetric deafness, of progressive progression during weeks or months (220). Both the innate immunity and the adaptive immune system are involved in the etiopathogenesis of the disease and are responsible for the histological changes observed in the cochlea of the patients affected with the autoimmune disease of the inner ear. These histological changes include Corti organ damage, neural degeneration, endolymphatic hydropsy, vascular stria lesion and osteogenesis, and cochlear basal loop fibrosis, endolymphatic sac fibrosis, and the presence of lymphocytes in the membranous labyrinth (221). Animal studies have shown the presence of autoantibodies and T cells against vestibulo-cochlear antigens (222). In addition, studies in humans revealed the presence of immune complexes in patients with the autoimmune disease of the inner ear (223). In only one study (151), hMSCs were used for the treatment of autoimmune disease-associated hearing loss. This study used hMSCs isolated from the adipose tissue and the mice as the experimental model. The auditory brainstem responses threshold and the cochlear morphology were the outcomes used by the study selected in this systematic review to assess the potential of hMSCs administration for the treatment of autoimmune hearing loss. Due to the fact that there were no human clinical trials among the studies selected, it was not possible to identify primary endpoints commonly used to evaluate the effectiveness of hMSCs administration for the treatment of autoimmune hearing loss in humans. However, in future clinical trials, specific endpoints such as the improvement in pure tone threshold and the improvement in speech discrimination should be used in conjunction to allow the evaluation of the effectiveness of hMSCs administration for the treatment of autoimmune hearing loss. In addition, exploratory endpoints such as the serum level of inflammatory cytokines and the proportion of inflammatory cells in the blood can be used to allow the researchers to identify what are the mechanisms responsible for the decrease in the pathological process observed after hMSCs administration. In the study selected, the administration of hMSCs improved the clinical parameters of the disease. Regarding the mechanisms of action proposed, Zhou et al. (151) demonstrated that the infusion of hMSCs decreased the proliferation of antigen-specific Th1 and Th17 cells and increased the production of the anti-inflammatory cytokine IL-10 in splenocytes. Additionally, administration of hMSCs also induced the generation of antigen-specific CD4^+^CD25^+^Foxp3^+^Treg cells. Table 8 summarizes the results obtained in the studies selected in this systematic review regarding the methodology employed and the effects of the administration of hMSCs for the treatment of autoimmune visual and auditory disorders.

**Table 8:** List of *in vivo* studies in which the therapeutic potential of the administration of hMSCs for the treatment of autoimmune visual and auditory disorders was evaluated.

**References**	**Autoimmune disease**	**Source of hMSC**	**Variables**	**Experimental model**	**Clinical effects**	**Mechanism proposed**
(163)	Autoimmune uveitis	Bone marrow	None	Mice	↓Histological disease score	↑MHC class II^lo^Ly6G^−^Ly6C^hi^CD11b^+^ cells in draining lymph nodes ↓CD4^+^ cell proliferation ↑CD4^+^ cell apoptosis ↓CD4^+^IFN-γ^+^ Th1 cells ↓CD4^+^IL-17^+^ Th17 cells
(81)	Autoimmune uveitis	Embryonic stem cells	None	Mice	↓Fundoscopic score ↓Histological disease score	↓T-cell proliferation ↓CD83 up-regulation in dendritic cells ↓IL-12 ↑CD4^+^CD25^+^FoxP3^+^ Treg cells
(150)	Autoimmune uveitis	Bone marrow	None	Mice	↓Autoimmune uveitis incidence ↓Histological disease score ↑Cumulative survival rate of corneal grafts	↑MHC classII^+^B220^+^CD11b^+^IL-10^+^ monocytes and macrophages with T-cell–suppressive activities
(151)	Autoimmune hearing loss	Adipose tissue	None	Mice	↓Auditory brainstem responses threshold ↓Cochlear pathology	↓Proliferation of antigen-specific Th1 cells ↓Proliferation of antigen-specific Th17 cells ↑IL-10 production in splenocytes ↑Generation of antigen-specific CD4^+^CD25^+^Foxp3^+^ Treg cells

*Both the methodology employed and the results obtained by each article are represented in this table*.

## Discussion

Through a statistical analysis of the data obtained in this systematic review, it was possible to determine which immune-related disease was more commonly treated by hMSCs administration among all articles analyzed. The results obtained have shown that in 29.4% of the articles selected hMSCs were administered for the treatment GvHD while in 19.8% of the articles they were used for the treatment of multiple sclerosis, in 15.4% for the treatment of Cronh's disease and ulcerative colitis and in 12.5% for the treatment of rheumatoid arthritis. Treatment of type I diabetes mellitus and systemic lupus erythematosus through the administration of hMSCs was also relatively common among the articles selected, with each of them accounting for 5.9% of all articles analyzed in this systematic review. Other immune-related diseases for which hMSCs were less commonly administered with therapeutic intentions include myasthenia gravis (2.2% of the articles), autoimmune lung fibrosis (1.5% of the articles), ankylosing spondylitis (0.73% of the articles), autoimmune hearing loss (0.73% of the articles), autoimmune uveitis (2.2% of the articles), hemophagocytic syndrome (1.5% of the articles), neuromyelitisoptica (0.73% of the articles), systemic sclerosis (0.73% of the articles) and type II refractory celiac disease (0.73% of the articles). However, a significantly higher proportion of articles in which hMSCs were administered for the treatment of GvHD were observed among the human studies selected, while the treatment of other immune-related diseases was significantly less common in this group. A total of 65.9% of the human studies selected administered hMSCs for the treatment of GvHD. This result emphasizes the need for the conduction of human clinical trials in which hMSCs are employed for the treatment of other important immune-related diseases.

In addition, it was possible to determine which sources were most commonly employed for the isolation of hMSCs that were used in the articles selected. In this context, the bone marrow, the umbilical cord and the adipose tissue represents the most common sources of hMSCs employed by the articles selected, with each of them accounting for 47.6, 20, and 18.6% of all the articles, respectively. Other sources of hMSCs less commonly used include menstrual blood (1.4% of the articles), dental pulp (3.5% of the articles), gingiva (1.4% of the articles), placenta and fetal membrane (3.4% of the articles) and embryonic stem cells differentiation (4.1% of the articles). Finally, statistical analysis of the data obtained though the evaluation of the articles selected allowed us to determine which experimental models were more commonly employed in the studies selected. The results obtained demonstrated that the majority of the studies selected (60.7%) have chosen mice as the experimental model while in 32.5% of the studies selected were conducted in humans. Other experimental models less commonly employed by the articles selected included rats (4.6% of the articles) and pigs (2.2% of the articles).

It should be notice that some immune-related diseases are more likely to be treated by hMSCs administration in the near future than others. Currently, the great majority of the clinical trials conducted evaluated the use of hMSCs administration for the treatment of GvHD. As a result, a much greater amount of information describing the clinical benefits and possible adverse effects of hMSCs administration for the treatment of GvHD exist. In fact, a formulation of *ex vivo* cultured hMSCs called Prochymal was recently developed by Osiris Therapeutics. This formulation is now available in a total of nine countries for the treatment of patients with steroid-resistant grade III and IV GvHD (20) and is currently being tested in a phase III clinical trial sponsored by Osiris Therapeutics. Other immune-related diseases that are likely to be treated by hMSCs administration in the near future include Crohn's disease, multiple sclerosis and type I diabetes mellitus. In one hand, the treatment of multiple sclerosis through hMSCs administration was assessed in several animal studies, and in a randomized placebo-controlled phase II clinical trial and in an open-label phase II study conducted by Llufriu et al. (134) and Bonab et al. (125), respectively. The treatment of Crohn's disease through hMSCs administration, on the other hand, was also evaluated in several animal studies and in a clinical phase I study conducted by Duijvestein et al. (116). Finally, the efficacy of hMSCs administration for the treatment of type I diabetes mellitus and the occurrence of side effects was assessed in several animal studies and in a phase I clinical trial conducted by Hu et al. (78). Despite being already tested in humans, administration of hMSCs for the treatment of hemophagocitic syndrome, neuromyelitis optica and celiac disease was conducted only in single case reports. Therefore, it is not likely that the treatment of these immune-related diseases will be conducted through hMSCs administration in the near future. The treatment of systemic sclerosis, ankylosing spondylitis and myasthenia gravis through hMSCs administration, despite already being tested in phase I clinical trials, still needs to be assessed in a higher number of studies before becoming a reality in clinical practice. In addition, other immune-related diseases such as systemic lupus erythematosus, rheumatoid arthritis, idiopathic pulmonary fibrosis, autoimmune uveitis and autoimmune hearing loss were only treated though hMSCs administration in animal models and, as a result, the treatment of these diseases through hMSCs administration is also not likely to become a reality in clinical practice in the near future.

Most of the studies analyzed in this systematic review reported positive results when hMSCs were administered for the treatment of immune-related diseases. Clinical effects commonly observed include an increase in the survival rates and a reduction in the severity and incidence of the immune-related diseases studied. In conjunction with this improvement of clinical parameters, many of the studies selected reported significant reduction in tissue pathology and inflammation following treatment with hMSCs. As a consequence, a decrease in the levels of several markers of the autoimmune process was also commonly observed. Furthermore, in the majority of the articles analyzed, an inhibition in the proliferation of inflammatory cells and a decrease in the infiltration of these cells into organs classically affected by the diseases studied was also reported. In general, hMSCs administration resulted in an inhibition in the proliferation and activation of cells from both innate and adaptive immunity, especially CD19^+^ B cells, CD4^+^ Th1 and Th17 cells, CD8^+^ T cells, NK cells, macrophages, monocytes and neutrophils. The clonal expansion of both Bregs and Tregs cells, however, was stimulated by the administration of hMSCs, as reported by many of the studies selected. Administration of hMSCs also resulted in a reduction in the levels of pro-inflammatory cytokines such as IFN-γ, TNF-α, IL-1, IL-2, IL-12, and IL-17 and in an increase in the levels of immunoregulatory cytokines such as IL-4, IL-10, and IL-13. Finally, the effects observed after the administration of hMSCs were also commonly attributed to the expression of molecules with immunoregulatory properties such as LIF, PD-L1, COX-2, activin A, TSG-6, HGF, PGE2, TGF-β1, FGF, IDO, VEGF, and HLA-G by these cells. However, as reported by some of the articles selected, some properties of the hMSCs administered such as the age and weight of the donor, the source and the number of passages might also affect significantly the potential of hMSCs for the treatment of immune-related diseases.

For instance, a study conducted by von Bahr et al. (49) demonstrated that GvHD patients who received early-passage MSCs had greater survival rates (75%) in comparison with patients who received late-passage MSCs (21%), 1 year after the beginning of the treatment. A study by Yu et al. (101) also demonstrated that the administration of late-passage hMSCs had a significantly compromised protective effect against mouse experimental colitis and that the production PGE2 and COX-2 by these cells was markedly reduced when compared to early-passage hMSCs. The influence of the weight of the donor in the immunoregulatory properties of adipose tissue-derived hMSCs was also analyzed in a study conducted by Strong et al. (136). This study showed that the administration of adipose tissue-derived hMSCs isolated from obese donors failed to alleviate clinical symptoms and inhibit inflammation in the central nervous system of murine experimental autoimmune encephalomyelitis, in contrast with the administration of adipose tissue-derived hMSCs isolated from lean subjects, which exhibited strong anti-inflammatory and therapeutic activities. Similarly, a study conducted by Scruggs et al. (121) demonstrated that experimental autoimmune encephalomyelitis mice treated with adipose tissue-derived hMSCs isolated from older donor cells had increased central nervous system inflammation and demyelination, when compared with the administration of adipose tissue-derived hMSCs isolated from younger donors.

Regarding the influence of the cell source in the immunomodulatory properties of hMSCs, a study conducted by Yamahara et al. (34) demonstrated that, in contrast to chorion-derived hMSCs administration, amnion-derived hMSCs administration markedly reduced T-lymphocyte proliferation and improved the pathological situation of GvHD mice though the production of PGE2 in higher quantities. In addition, a study conducted by Luz-Crawford et al. (72) demonstrated that, while the administration of menstrual blood-derived hMSCs was able to increase the survival of GvHD mice, bone marrow-derived hMSCs administration did not. In contrast, this study has also shown that only the administration of bone marrow-derived hMSCs resulted in a potent therapeutic effect in mice with collagen induced arthritis, while menstrual blood-derived hMSCs administration did not. A study conducted by Wang et al. (102), however, demonstrated that the administration of hMSCs obtained from the differentiation of embryonic stem cells exerted both preventive and therapeutic effects on experimental autoimmune encephalomyelitis, while bone marrow-derived hMSCs exhibited significantly lower therapeutic efficacy. Furthermore, a study by Payne et al. (128) compared the therapeutic potential of the administration hMSCs isolated from bone marrow, umbilical cord and adipose tissue for the treatment of experimental autoimmune encephalomyelitis. This study demonstrated that the administration of adipose tissue-derived hMSCs had the most significant impact on clinical and pathological disease outcomes, while bone marrow-derived hMSCs administration resulted in a negligible effect on the disease course. Administration of umbilical cord-derived hMSCs had also a positive and significant impact in the progression of the disease. Moreover, a study conducted by Santos et al. (95) demonstrated that the administration umbilical cord-derived hMSCs can reduce paw edema *in vivo* more efficiently than bone marrow-derived hMSCs in an acute carrageenan-induced arthritis model. Finally, a study by Liu et al. (119) showed that the administration of the supernatant from a bone marrow-derived hMSC culture, which expressed higher level of TGF-β1, has a better therapeutic efficacy in improving the survival rate and reducing pulmonary inflammation and fibrosis in a bleomycin-induced pulmonary fibrosis mouse model when compared to umbilical cord-derived hMSCs, which secrete a lower level of TGF-β1.

Stimulation of hMSCs with immunosuppressive drugs prior to administration had also a significant effect in the efficacy of these stem cells in the treatment of immune-related diseases, as reported by some of the articles selected. For instance, a study conducted by Kim et al. (40) demonstrated that the administration of adipose tissue-derived hMSCs previously treated with rapamycin resulted in a greater reduction in the severity of aGvHD, when compared with the administration of untreated adipose tissue-derived hMSCs. It was also demonstrated by this study that this improvement in the therapeutic potential observed after the administration of adipose tissue-derived hMSCs previously treated with rapamycin was associated with a reduction in the number of Th17 cells and an increase in the number of Tregs cells. Furthermore, a study conducted by Girdlestone et al. (61) demonstrated that rapamycin-treated but not untreated umbilical cord-derived hMSCs significantly inhibit the onset of GvHD in mice. On the other hand, a study conducted by Liu et al. (114) demonstrated that the stimulation of dental pulp stem cells with acetylsalicylic acid upregulates TERT/FASL signaling in these cells, resulting in an increase in T-cell apoptosis and in the improvement in the clinical parameters of dextran sodium sulfate induced colitis in mice.

Some of the studies selected also reported positive results when hMSCs were stimulated with inflammatory cytokines prior to their administration for the treatment of immune-related diseases. For instance, a study conducted by Torkaman et al. (144) demonstrated that the administration of umbilical cord-derived hMSCs previously treated with IFN-γ resulted in a higher inhibition in leukocyte infiltration into the central nervous system and in the reduction in the symptoms of experimental autoimmune encephalomyelitis in mice, compared to the administration of untreated umbilical cord-derived hMSCs. Furthermore, administration of umbilical cord-derived hMSCs previously treated with IFN-γ stimulated the clonal expansion of Treg cells and decreased the secretion and gene expression of inflammatory cytokines in experimental autoimmune encephalomyelitis mice. In addition, a study conducted by Tobin et al. (35) demonstrated that bone marrow-derived hMSCs, when stimulated by IFN-γ, reduced aGVHD-related weight loss and pathology, while greatly increasing the survival time of mice with aGVHD. Similarly, a study conducted by Duijvestein et al. (109) demonstrated that the administration of bone marrow-derived hMSCs pretreated with IFN-γ resulted in an increase in the survival rates and in the attenuation in the development and in the symptoms of dextran sodium sulfate and trinitrobenzene sulfonate induced colitis in mice, in contrast with the administration of non-stimulated bone marrow-derived hMSCs. Administration of bone marrow-derived hMSCs pretreated with IFN-γ also resulted in a significant reduction in serum amyloid A protein levels and local proinflammatory cytokine levels (especially Th1 cytokines) in colonic tissues. Finally, hMSCs pretreated with IFN-γ showed higher migration potential than unstimulated MSCs to sites within the inflamed intestine.

With the intention of enhancing the immunomodulatory properties of hMSCs, in some of the studies selected, hDSCs were transduced with several distinct genes and applied for the treatment of immune-related diseases. In a study conducted by Sadeghi et al. (33), for example, dental pulp stem cells were transduced with immunosuppressive genes encoding IL-10, PGE2 receptor, IDO, IFN-γ, and PDL-1 proteins. However, no difference was observed between transduced and untransduced dental pulp stem cells in both *in vitro* experiments and for the treatment of GvHD mice. Additionally, a study conducted by Sun et al. (74) demonstrated that the transplantation of adipose tissue-derived hMSCs overexpressing betatrophin into mice with streptozotocin-induced diabetes ameliorated the hyperglycemia and weight loss associated with the disease and also significantly enhanced the ratio of β-cells per islet compared to the transplantation of adipose tissue-derived hMSCs alone. Furthermore, a study conducted by Liao et al. (138) demonstrated that the transfection of bone marrow-derived hMSCs with PSGL-1/SLeX mRNA enhanced the homing of these stem cells to the inflamed spinal cord and, as a consequence, resulted in superior therapeutic function over native bone marrow-derived hMSCs, as evidenced by significantly improved myelination and decreased lymphocytes infiltration into the white matter of the spinal cord of mice with experimental autoimmune encephalomyelitis. In addition, a study conducted by Choi et al. (86) showed that the administration of adipose tissue-derived hMSCs overexpressing the anti-CTLA4 gene protected against the destruction of cartilage in mice with collagen-induced arthritis. This protective effect was more effective when adipose tissue-derived hMSCs overexpressing the anti-CTLA4 gene were administered, compared to the administration of adipose tissue-derived hMSCs alone. As expected, the serum levels of type II collagen autoantibodies and C-telopeptideof type II collagen were also significantly lower in the group transplanted with adipose tissue-derived hMSCs overexpressing the anti-CTLA4 gene, while the ratio of Treg/Th17 cells was increased, compared with the group treated with adipose tissue-derived hMSCs alone. Finally, in a study conducted by Park et al. (92), bone marrow-derived hMSCs were transfected with recombinant minicircles encoding etanercept and applied for the treatment of collagen-induced arthritis in mice. This study demonstrated that arthritis subsided more efficiently in collagen-induced mice injected with bone marrow-derived hMSCs transfected with recombinant minicircles encoding etanercept than in those injected with conventional bone marrow-derived hMSCs or etanercept only.

Lastly, in some of the studies selected, other types of therapies were applied in conjunction with hMSCs administration for the treatment of immune-related diseases. For instance, a study conducted by Hou et al. (126) demonstrated that the administration of bone marrow-derived hMSCs combined with minocycline resulted in a greater reduction in clinical scores, along with the attenuation of inflammation, demyelination, and neurodegeneration in experimental autoimmune encephalomyelitis mice, compared to the use of minocycline or bone marrow-derived hMSCs alone. In addition, the combined treatment also resulted in a significant decrease of the number of apoptotic cells, compared with either treatment alone. Finally, a study conducted by Im et al. (59) demonstrated that, compared with single cell therapy, the administration of adipose tissue-derived hMSCs combined with Tregs cells resulted in a higher reduction in the mortality rates and increased the engraftment rate and the donor-specific tolerance to skin allografts across full major histocompatibility complex barriers in GvHD mice, through reciprocal regulation of Treg/Th17 cells.

## Final considerations

In this systematic review, the treatment of many types of immune-related diseases was conducted through the administration of hMSCs. Positive results were usually reported and attributed to the paracrine effects of molecules secreted by hMSCs on immune cells. However, while a significant amount of the studies selected were conducted in humans, in the majority of them, animal models of immune-related diseases were used. This emphasizes the need for the conduction of randomized clinical trials in which the potential of hMSCs for the treatment of relevant immune-related diseases. Immune-related diseases that are likely to be treated by hMSCs administration in the future include GvHD, Crohn's disease, multiple sclerosis and type I diabetes mellitus. Other diseases such as hemophagocitic syndrome, neuromyelitis optica, celiac disease, systemic sclerosis, ankylosing spondylitis, myasthenia gravis, systemic lupus erythematosus, rheumatoid arthritis, idiopathic pulmonary fibrosis, autoimmune uveitis and autoimmune hearing loss either lacks more studies in general or randomized clinical trials and are not likely to be treated through hMSCs administration in the near future. It is also important to determine what is the most appropriate source of hMSCs that should be applied for the treatment of each immune-related disease. The elaboration of strategies for the enhancement of the immunomodulatory properties of hMSCs is also relevant and was proposed by some of the articles selected, as previously discussed. In conclusion, despite the need for further studies, the treatment of immune-related diseases through the administration of hMSCs is progressively ceasing being only a promising possibility and becoming a reality.

## Author contributions

The entirety of the manuscript was written by AL. All figures and tables were also designed by AL. The process of screening of titles and abstracts for the inclusion or exclusion of articles for this systematic review was conducted by both AL and CP. Disagreements during this process were resolved by discussion with DB. Finally, both DB and MA were responsible for the revision of the manuscript before submission.

### Conflict of interest statement

The authors declare that the research was conducted in the absence of any commercial or financial relationships that could be construed as a potential conflict of interest.
